# “*And* *if you gaze long into an abyss, the abyss gazes also into thee*”: four morphs of Arctic charr adapting to a depth gradient in Lake Tinnsjøen

**DOI:** 10.1111/eva.12983

**Published:** 2020-06-26

**Authors:** Kjartan Østbye, Marius Hagen Hassve, Ana‐Maria Peris Tamayo, Mari Hagenlund, Thomas Vogler, Kim Præbel

**Affiliations:** ^1^ Faculty of Applied Ecology, Agricultural Sciences and Biotechnology Inland Norway University of Applied Sciences Campus Evenstad Norway; ^2^ Department of Biosciences Centre for Ecological and Evolutionary Synthesis (CEES) University of Oslo Oslo Norway; ^3^ Faculty of Biosciences, Fisheries and Economics Norwegian College of Fishery Science UiT Arctic University of Norway Tromsø Norway

**Keywords:** adaptive radiation, ecological speciation, microsatellites, morphs, mtDNA, natural selection, niche specialization, Pleistocene ice age, population divergence, *Salvelinus alpinus*

## Abstract

The origin of species is a central topic in biology. Ecological speciation might be a driver in adaptive radiation, providing a framework for understanding mechanisms, level, and rate of diversification. The Arctic charr *Salvelinus alpinus* L. is a polymorphic species with huge morphological and life‐history diversity in Holarctic water systems. We studied adaptive radiation of Arctic charr in the 460‐m‐deep Lake Tinnsjøen to (a) document eco‐morphology and life‐history traits of morphs, (b) estimate reproductive isolation of morphs, and (c) illuminate Holarctic phylogeography and lineages colonizing Lake Tinnsjøen. We compared Lake Tinnsjøen with four Norwegian outgroup populations. Four field‐assigned morphs were identified in Lake Tinnsjøen: the planktivore morph in all habitats except deep profundal, the dwarf morph in shallow‐moderate profundal, the piscivore morph mainly in shallow‐moderate profundal, and a new undescribed abyssal morph in the deep profundal. Morphs displayed extensive life‐history variation in age and size. A moderate‐to‐high concordance was observed among morphs and four genetic clusters from microsatellites. mtDNA suggested two minor endemic clades in Lake Tinnsjøen originating from one widespread colonizing clade in the Holarctic. All morphs were genetically differentiated at microsatellites (*F*_ST_: 0.12–0.20), associated with different mtDNA clade frequencies. Analyses of outgroup lakes implied colonization from a river below Lake Tinnsjøen. Our findings suggest postglacial adaptive radiation of one colonizing mtDNA lineage with niche specialization along a depth–temperature–productivity–pressure gradient. Concordance between reproductive isolation and habitats of morphs implies ecological speciation as a mechanism. Particularly novel is the extensive morph diversification with depth into the often unexplored deepwater profundal habitat, suggesting we may have systematically underestimated biodiversity in lakes. In a biological conservation framework, it is imperative to protect endemic below‐species‐level biodiversity, particularly so since within‐species variation comprises an extremely important component of the generally low total biodiversity observed in the northern freshwater systems.

## 1 INTRODUCTION

Revealing processes behind adaptive diversity, and formation of species, are central themes in evolutionary biology. Although studied for a long time, the mechanisms for adaptive radiation and speciation appear enigmatic. Our consensus understanding is that adaptive radiation by natural selection has been important in the origin of populations and species (Darwin, 1859;Mayr, 1942;Schluter, 2000). In a biological conservation framework, we should center less on species moving toward protecting biological diversity below the species level, which reflects ongoing natural (non)adaptive speciation processes. The low aquatic species diversity in the north means that the within‐species variation is an extremely important component of the total biodiversity (Chavarie, Howland, Harris, & Tonn, 2015;Fraser, Weir, Bernatchez, Hansen, & Taylor, 2011;Moore et al., 2014;Reist, Power, & Dempson, 2013). Thus, the speciation process as a fundamental question in evolutionary biology has also important and practical relevance in applied biological conservation (Coates, Byrne, & Moritz, 2018).

Scientists continuously search for ideal study systems and species groups, to illuminate how speciation processes are acting under evolutionary scenarios and timescales. Here, highly recognized model species used as rewarding looking‐glasses into the species‐formation process comprise, for example, Darwin's finches on the Galapagos Islands, European‐Mediterranean sparrows, the *Anolis* lizards, cichlid fishes, the threespined stickleback, and sunflowers (Grant & Grant, 2008;Hermansen et al., 2011;Miller, Rosti, & Schluter, 2019;Moyers & Rieseberg, 2016;Salazar, Castañeda, Londoño, Bodensteiner, & Muñoz, 2019;Salzburger, 2018). The polymorphic northern freshwater fishes of *Coregonus* and *Salvelinus* species complexes are becoming increasingly recognized as good model systems in this regard (Bernatchez, 2004;Jonsson & Jonsson, 2001;Klemetsen, 2010). Speciation is a complex issue (e.g., Wilkins, 2018), where the theoretical–empirical framework presents avenues for adaptive diversification in speciation (Gavrilets, 2004;Seehausen & Wagner, 2014;Suzuki & Chiba, 2016). Across examples of adaptive radiation, similarities exist for patterns and processes, where one could tailor models specifically to each species system to derive an understanding of mechanisms by empirically parameterizing theoretical models (Gavrilets & Vose, 2007;Thibert‐Plante et al., 2020). The insight from theoretical–empirical analyses can point toward important areas where we need to fill knowledge gaps that surface through predictive theoretical models when attempting to add empirical values.

In the ice‐covered northern Eurasian hemisphere, the late Pleistocene ice sheet set the frame for colonization and postglacial adaptation to lakes as the maximum extent of the ice sheet occurred at ca. 21, 000 years before present (ybp) and deglaciation at ca. 10–20, 000 ybp (Hughes, Gyllencreutz, Lohne, Mangerud, & Svendsen, 2016;Mangerud et al., 2004;Patton et al., 2017). The Pleistocene ice age started ca. 2.58 million years before present, with alternating phases of glaciation (of roughly 70, 000–100, 000 years’ duration) and interglacials (10, 000–30, 000 years’ duration) (Andersen & Borns, 1994;Lorens, Hilgen, Shackelton, Laskar, & Wilson, 2004;Rapp, 2015). The Pleistocene ice age dynamics represents a long time series where flora and fauna likely repeatedly colonized new land and retracted to glacial refugia. Such conditions created opportunities for allopatric differentiation, secondary contact, and sympatric diversification among and within species (Hewitt, 2004;Swenson & Howard, 2005;Taberlet, Fumagalli, Wust‐Saucy, & Cosson, 1998). Thus, Holarctic lakes comprise a unique window into the adaptive diversification process of colonizing Arctic charr (*Salvelinus alpinus*, L) where the degree and rate of novel, or parallel adaptations, can be studied by contrasting old versus young glacial geological systems represented by genetic lineages and carbon‐isotope‐dated lakes. Ecological opportunity for diversification via intraspecific competition and niche radiation in species‐poor postglacial lakes may be an important mechanism in morph and species formation in several fish taxa (Robinson & Wilson, 1994;Seehausen & Wagner, 2014;Siwertson et al., 2010). One mechanism that could build up reproductive isolation as a secondary product is termed ecological speciation (Hendry, 2009;Rice, 1987) and could have been central in adaptive proliferation of morphs into all lake niches. With regard to sympatric Arctic charr morphs, several evolutionary scenarios are hypothesized (see also Seehausen & Wagner, 2014). First, the lake could have been colonized by divergent genetic lineages (associated with different morphs) coming into secondary contact after separation for thousands of years in glacial refugia. Secondly, sympatric morphs may represent a real intralake sympatric adaptive diversification after colonization of one genetic lineage (comprising one initial ancestral morph). Thirdly, a combination of such scenarios could have occurred, generating temporal dynamics in gene pool sharing via expansion–contraction, adaptive divergence, speciation reversal, introgression and hybrid swarm dynamics, and subsequent divergence based on novel combinations of genetic variants to be selected upon. Under such adaptive diversification mechanisms, also genetic drift and phenotypic plasticity may be important processes (Häkli, Østbye, Kahilainen, Amundsen, & Præbel, 2018;Seehausen & Wagner, 2014;West‐Eberhard, 1989).

The highly polymorphic Arctic charr species complex has a Holarctic distribution and is the most cold adapted northern freshwater fish species, where some populations are anadromous, while most populations are stationary in freshwater (Klemetsen, 2010;Taylor, 2016). Arctic charr occupy species‐poor Holarctic lakes, suggesting ecological opportunity for adaptive radiation into available niches (Klemetsen, 2010;Knudsen, Klemetsen, Amundsen, & Hermansen, 2006). Many Arctic charr lakes apparently only harbor a generalist morph, supported by the relative few studies revealing polymorphism. Some of these monomorphic populations, with a generalist morph, utilize both littoral and pelagial habitats through ontogenetic habitat shifts (Klemetsen, 2010). In a much fewer set of lakes, two more or less distinct morphs, for example, a littoral and a pelagic morph, may co‐occur (Hooker et al., 2016;Westgaard, Klemetsen, & Knudsen, 2004), suggesting lake‐specific temporal persistence of niches for the evolution and coexistence of two different morphs. In a very few lakes, a third morph is found in the profundal, termed the profundal morph, coexisting with, for example, the littoral and pelagic morph (Moccetti et al., 2019;Skoglund, Siwertsson, Amundsen, & Knudsen, 2015). Only in one single lake worldwide, namely Lake Thingvallavatn in Iceland, four sympatric morphs are reported having radiated into all lake niches: a small and large benthic morph, a pelagic morph, and a piscivore morph (Jonsson et al., 1998). Arctic charr morphs that adapt to divergent niches may show parallelism among lakes with independent origin of morph pairs (Gordeeva, Alekseyev, Matveev, & Samusenok, 2015). Here, similar morphs can evolve through parallel or nonparallel evolutionary routes revealing similar gene expression as seen in independently derived morph replicates of two genetic lineages (Atlantic and Siberian lineage) in Arctic charr (Jacobs et al., 2020). This suggests the presence of a highly robust adaptive system in the Arctic charr complex for deriving the same evolutionary outcome from different genetic starting points (historical contingency: adaptive standing genetic diversity, genomic architecture) as response to similar selection pressures. However, there are often lake‐specific differences in morph variance in, for example, niche occupation, phenotype, and life history (Knudsen, Amundsen, Primicerio, Klemetsen, & Sørensen, 2007;Moccetti et al., 2019). This large‐scale parallel evolution in Holarctic lakes, with similar morphs appearing, is a unique feature when studying natural selection and early stages in the speciation continuum, making the Arctic charr species complex an excellent model system in evolutionary biology and eco‐evo‐devo studies.

BOX 1 1 We got involved with this nice man named Louis many years back during our own PhD (Kjartan) and PostDoc work (Kim), being kindly invited to his lab in Quebec for collaboration. We were not there at the same time, but Louis and we shared the same love to studies of adaptive radiation and ecological speciation in Coregonus (of course!). We worked on understanding evolutionary and genetic patterns and processes underlying the vast phenotypic and genotypic variation found in the European whitefish complex. A daunting and life‐long task, that we never would have been able to advance if not for the tremendous contribution and insights from Louis, especially from the Lake whitefish crossings, and his pioneering work on enabling and using genomic tools in non‐model species. We also still remember our discussions a late evening in 2012 in Mondsee, Austria, where you encouraged us to undertake this study in Arctic charr! Based on our long‐term friendship it is evident that Louis is a strong scientific person, but he has not traded off important ordinary down‐to‐earth traits such as good mood, being able to party, going fishing and hunting. Particularly, his strong social side is an essential positive trait to mention, as Louis has run his lab as an integrated social unit where the atmosphere is relaxing, and competitive, and based on a curiosity‐driven mindset. In such a rewarding environment, filled with top‐notch personnel and state of the art technologies, even untrained naive hillbilly‐rascals from Norway and Denmark were able to learn fast and efficiently. Louis has the brilliant ability to really listen to his students and colleagues, and indeed a special nose for cutting‐edge studies that needs to be conducted for the common good for the scientific society. Louis has been very influential for both of us with regard to our mind‐sets in our scientific careers, and as a friend, colleague and collaborator in our scientific projects. We are indeed very fond of this Basque‐Quebecois‐Canadian guy and look forward to the years to come.

Here, we report on a new Arctic charr system harboring a striking diversity in phenotypes and life histories, apparently associated with a depth–temperature–productivity–pressure gradient in the 460‐meter‐deep oligotrophic Lake Tinnsjøen in Norway (Box 1). The history before our study is as follows. In 1944, in the occupied Norway during the Second World War, the Norwegian partisans sunk the railway ferry *D/F Hydro* carrying an estimated 20 barrels with 500 kilo of heavy water (D_2_O) in Lake Tinnsjøen. The German occupation government had the purpose to construct an atomic bomb back home in Germany using D_2_O (Dagbladet, 2018;National Geographic, 2018). It has been debated whether this Second World War famous sabotage action hampered or stopped Hitler's attempt to produce the atomic bomb. Almost 50–60 years later, in 1993 and 2004, a Norwegian team on their search for the sunken ferry, making a Second World War news report regarding the presence of heavy water on the ferry, was able to locate it at 430 meters depth using a ROV submarine. At the same time, they also observed small fish residing at the bottom. The team successfully retrieved two fish specimens that were later classified as Arctic charr (Søreide, Dolmen, & Hindar, 2006). The knowledge about the Arctic charr diversity within Lake Tinnsjøen up to that date comprised a study by Hindar, Ryman, and Ståhl (1986) showing that a dwarf and planktivore morph grouped together (being statistically different from each other) compared to yet other Norwegian lakes when analyzing allozymes. From old age, local fishermen in Lake Tinnsjøen have recognized a rare deepwater morph of Arctic charr locally named “Gautefisk” (“Gaute” is a Norwegian male name, and “fisk” is fish in Norwegian). This morph has different coloration from other morphs in the lake, and different body proportion, weighing up to 4–6 kg (Brabrand, 1994). Thus, when summarizing available information, a set of four morphs were suggested in Lake Tinnsjøen.

As no progress occurred considering scientific studies on the small white fish from the bottom of the lake from the ROV team, we conducted a fish survey in the lake in 2013 to document the occurrence of morphs. We set up three main research topics with regard to the Lake Tinnsjøen Arctic charr diversity: (a) to document eco‐morphology and life‐history traits (body shape, proportional catch in habitat, age, weight) of field‐assigned morphs, (b) to estimate reproductive isolation of field‐assigned morphs or fish assessed using unbiased methods (microsatellites), and (c) to illuminate the phylogeography and ancestral lineages colonizing Lake Tinnsjøen (mtDNA‐CytB sequences). To accomplish these tasks, we collected fish in different habitats in the pelagial, littoral, shallow‐moderate profundal and in the deep profundal. In the field, we classified fish to morphs from exterior phenotype, while in the laboratory, we assessed morphological (body shape) and genetic divergence using mtDNA and nDNA markers. We further performed a Holarctic phylogeography retrieving online genetic sequences to evaluate lineages colonizing Lake Tinnsjøen. The strength of association of field‐assigned morphs and genetically identified morphs using microsatellites (i.e., genetic clusters or populations) was tested. We compared mtDNA and nDNA in Lake Tinnsjøen with four Norwegian outgroup lakes. Using a putative ancestor below in the same drainage, we compared body shape to the Lake Tinnsjøen morphs.

## 2 METHODS

### 2.1 Material used for different analyses

The material used for the different analyses is summarized in Appendix S1: Table S1.

### 2.2 Study area, fish sampling, and field‐assigned morphs

**FIGURE 1:**
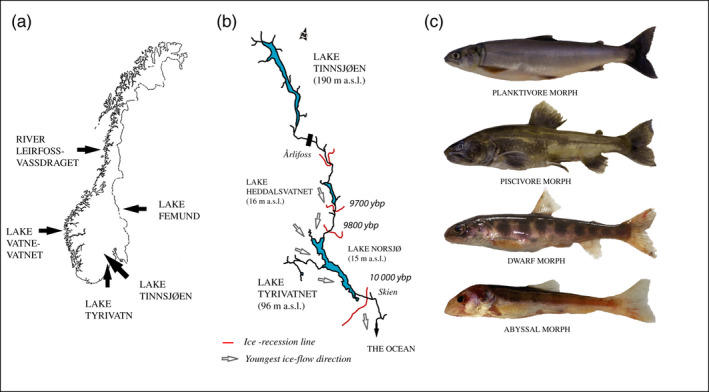
(a) Norway with Lake Tinnsjøen and the four outgroups sampled. (b) River Skiensvassdraget wherein Lake Tinnsjøen is situated. Red lines denote dated ice‐recession lines in years before present (ybp) from Bergstrøm (1999). Gray arrows denote the youngest ice‐flow direction in the end of the Pleistocene glaciation from Bergstrøm (1999). The black bar indicates the upper deposits of marine sediments. (c) The four nominal field‐assigned Arctic charr morphs (FA‐morphs) observed within Lake Tinnsjøen (note: fish scaled to the same length)

Lake Tinnsjøen (60 38 15.6 N, 11 07 15.2 E) is a long (35 km), large (51.38 km^2^), and deep (max depth of 460 m, 190 m mean depth) oligotrophic lake in southeastern Norway (Figure 1a,b) (NVE, 1984). High mountain sides surround the lake descending steeply into the lake resulting in a relatively small littoral area compared to an extensive pelagic volume and a large profundal area. In the southern and northern ends of the lake, larger littoral areas exist. The littoral zone is exposed to the elements such as wind and waves. The shoreline is monotonous with few bays and only two small islands. The littoral zone is composed mostly of bedrock, large boulders, smaller rocks, and sand in less exposed areas and in the deeper layers. The pelagic zone is extensive. The profundal appears to differ structurally in shallow and deep areas—composed of bedrock, boulders, sand, and larger‐sized organic matter in shallow areas, while more fine particulate organic detritus dominates in the deep profundal areas (based on organic matter on catch equipment and from videos by the Norwegian Broadcasting Company (www‐link; no longer valid)). A survey in Lake Tinnsjøen in June 2006 by Boehrer, Golmen, Løvik, Rahn, and Klavness (2013) gave an oxygen concentration of 11.5–12.0 mg/L from surface down to 460 m depth, a temperature profile from 4.0 to 3.3°C from 50 to 460 meters depth, conductivity of 10.0–8.0 µS/cm from 0 to 460 m depth, and dissolved oxygen ranging from 90% to 85% from 0 to 460 m depth. Lund (1948) sampled Lake Tinnsjøen once a month from December 1946 to December 1947 and found that below ca 80 m depth, the temperature was at a constant 4°C (depth stratified), while warming up to ca. 18–20°C in top layer in summer. Thus, Lake Tinnsjøen offers a divergent temperature profile (and light, pressure, and productivity in habitats, depths, and niches) in pelagic and littoral–benthic depth gradients from surface to 460 m.

We collected Arctic charr from Lake Tinnsjøen during 2013 and from four additional Norwegian outgroup populations (see below) north, west, east, and south of Lake Tinnsjøen in 2013–2015 (Figure 1a). Fish were caught in four lake habitats (can be viewed as crude nominal niches for individuals and morphs) in Lake Tinnsjøen using equipment described below. At this stage, we do not reveal the exact sampling sites until the taxonomic status of the new abyssal morph has been described and conservation biology authorities in Norway have considered the situation with regard to its conservation value. Particularly relevant here are the population size and uniqueness of the new discovered morph, and what conservation status it merits. As the lake has steep mountain sides entering the lake, it is hard to place equipment precisely at predetermined positions. Thus, habitat and depth ranges fished were grouped to be able to compare catch among four nominal lake habitats. The four lake habitats (nominal niches) sampled (and defined by us) in Lake Tinnsjøen in 2013 were as follows: (a) the pelagial (gillnets at < 20 m depth, in areas with depths of > 30 m, and > 50 meters from the shore), (b) the littoral (gillnets from shore < 20 m depth), (c) the shallow‐moderate profundal (gillnets, traps, and hook and line from shore at > 20 m and < 150 m depth), and (d) the deep profundal (traps at > 150 m depth, >100 m from the shore).

Sampling was conducted with gillnets, baited anchored longlines, and traps. Initially, we aimed at fishing with a standardized effort **x** equipment in all niches, but due to the experimental nature of fishing Arctic charr at depths > 150 m, and the low fish density, it was difficult to obtain sufficient sample sizes. Thus, we intensified the effort in the different habitats with the catch methods that worked best. As such, the material obtained may not be fully representative of fish populations at all depths and habitats, but represents an opportunistic sampling strategy under quite challenging fishing conditions. We used different monofilament series coupled in gangs when fishing with gillnets. In the pelagial, we used a 12‐panel multimesh Nordic series (each net: 6 × 60 m) with mesh sizes (in the following order) of 43, 19.5, 10.0, 55.0, 12.5, 24, 15.5, 35.0, 29.0, 6.3, 5.0, and 10.0 mm (knot to knot) and extended Jensen floating series (each net: 6 × 25 m) with mesh sizes of 13.5, 16.5, 19.5, 22.5, 26.0, 29.0, 35.0, 39.0, 45.0, and 52.0 mm. In the littoral, we used extended Nordic and Jensen littoral net series (each net: 1.5 × 60 m or with the same mesh size as in the pelagic zone) including extra nets of some of the largest meshes. We used traps at 20–60 m depth, and Jensen littoral net series (see above for specifications) and hook and line down to 150 m depth in the shallow‐moderate profundal. In the deep profundal, we used traps baited with cheese at 150–350 m depth. The baited anchored longlines (ca 220 m long; 3–4 mm line; 180 hooks; size 1, 1/0, and 2), aimed at catching piscivorous Arctic charr, were placed vertically close to the shoreline (<100 m) and in a few cases horizontally at the bottom. As these attempts resulted in a low catch, the hook and line approach was not used extensively. Nets and baited lines were checked after 12 hr, and traps could be out for 48 hr. A motorized winch was used for hauling equipment. All catch was grouped in lake habitats (nominal niches) despite different types of gear used. A total effort of 42 Nordic multimesh and 225 Jensen‐net nights, 1,001 trap nights, and 27 line nights were implemented in fishing. Besides Arctic charr, we caught brown trout, perch (*Perca fluviatilis*), and Eurasian minnow (*Phoxinus phoxinus*) (catch statistics not reported as being minute, <10 fish in few locations). The lake only holds the four fish species. The Eurasian minnow was introduced in Lake Tinnsjøen recently (1960–1970s).

Fish were killed using an overdose of benzocaine and transported dead on ice to the field laboratory at Lake Tinnsjøen. In the field, all the fish were subjectively assigned to four nominal morphs based on exterior morphology: (a) planktivore, (b) dwarf, (c) piscivore, and (d) abyssal (see representative individuals in Figure 1c). Each fish was classified as one of the four morphs despite variation within morphs and uncertainties. This field assignment of morphs was labeled as field‐assigned morphs (hereafter FA‐morphs). Length and weight were recorded, with sex and maturity stage, and age from otoliths in the laboratory. A DNA sample was taken in the field and stored on 96% EtOH for use in analyses (description below).

The four additional Norwegian outgroup populations of Arctic charr were situated to the north (River Leirfossvassdraget; anadromous sea‐running), west (Lake Vatnevatnet), east (Lake Femund), and south (Lake Tyrivatn) of Lake Tinnsjøen (Figure 1a). The three latter Arctic charr populations were stationary in freshwater. The sampling equipment, effort, and placement varied among lakes comprising gillnets with at least 16.5, 19.5, 22.5, and 29.0 mm (knot to knot) and/or modified Jensen series or Nordic multimesh panels set in littoral, pelagic, and profundal areas. In the laboratory, these four outgroup populations were analyzed as described above for Lake Tinnsjøen. A DNA sample was stored in 96% EtOH for genetic analyses. These four populations were used as selected outgroups in microsatellite analyses, in mtDNA‐based phylogenetic analyses, and partly in the morphological analyses. Arctic charr in Lake Tyrivatn was inferred as a putative “ancestral state” founder that could have colonized Lake Tinnsjøen, and was thus used for comparative purposes in microsatellite, mtDNA, and morphometric analyses (Figure 1a,b). This was anticipated as the lake is situated far below Lake Tinnsjøen in the same water system (see argumentation of likely colonization route in discussion). The real founding population into Lake Tinnsjøen is currently unknown.

### 2.3 Eco‐morphological and life‐history trait divergence in the charr morphs

**FIGURE 2:**
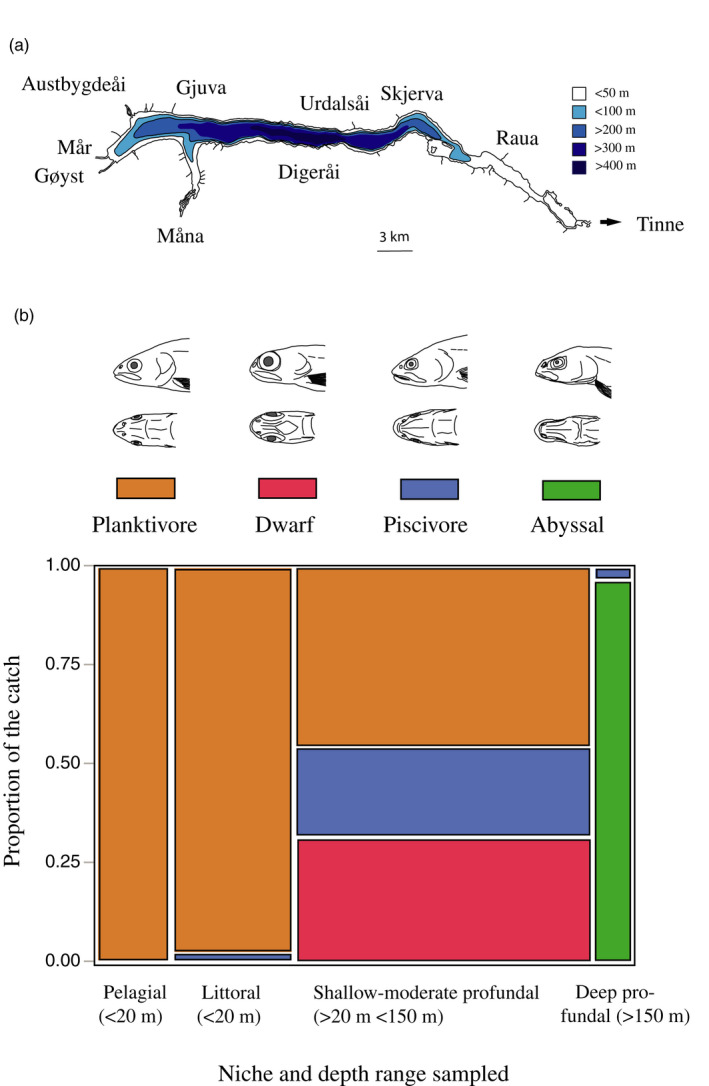
(a) A crude bathymetric map of Lake Tinnsjøen (modified from The Norwegian Water resources and Energy Directorate; http//gis3.nve.no/metadata/tema/DKBok1984/Dybdekart_1984.htm) (NVE, 1984). (b) Association between the catch of the four FA‐morphs in the four lake habitats in Lake Tinnsjøen. A drawing of representative heads (lateral and ventral views) of each of the four FA‐morphs is given in the top panel

In Lake Tinnsjøen, the association between habitat occurrence and FA‐morphs was tested using χ^2^ statistic in JMP 11.2 (SAS institute Inc, 2013). See bathymetric map in Figure 2a. The main purpose here was to reveal the association between FA‐morphs and habitat at catch; however, we are aware of the putative bias in having used different fishing gear in different habitats.

**FIGURE 3:**
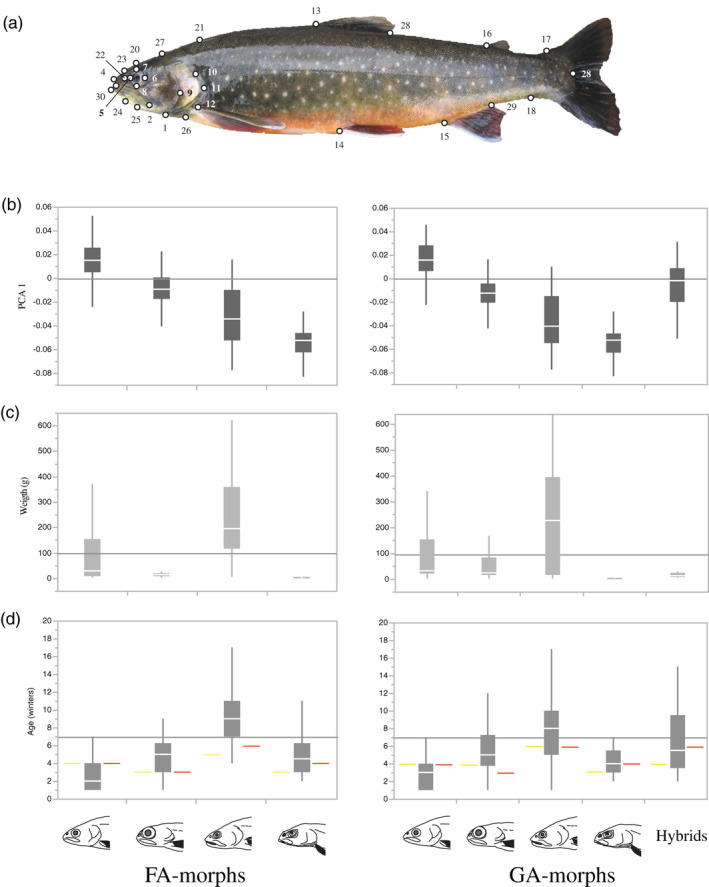
(a) The 30 landmarks used for body shape analyses in Lake Tinnsjøen: 1. lower edge of preoperculum, 2. edge of maxillary bone, 3. mouth opening, 4. tip of snout, 5.–8. eye positions, 9. mid‐edge of preoperculum, 10. posterior edge of preoperculum, 11. posterior edge of operculum, 12. pectoral fin, 13. and 28. dorsal fin, 14. pelvic fin, 15. and 29. anal fin, 16. adipose fin, 17. upper tail root, 18. lower tail root, 19. end of the side line organ, 20. top of head, 21. back above pectoral fin, 22. nostril, 23. over nostril, 24, under‐jaw, 25. edge of mouth, 26. lower edge of operculum, 27. transition zone from head to body, and 30. edge of lower lip. (b) Principal component axis 1 versus respectively FA‐morphs (left panel) and GA‐morphs (right panel) based on the 30 landmarks. (c) Weight versus FA‐morphs and GA‐morphs. (d) Age versus FA‐morphs and GA‐morphs. The youngest sexually mature male (yellow line) and female (red line) are given. The graphs denote median values (white horizontal line), the 25% to 75% percentiles (solid blocks), and the 10% to 90% percentiles (gray vertical line). In figure a–c, arbitrarily selected horizontal lines have been imposed for helping out visual comparisons among the four FA‐morphs and the four GA‐morphs, and in two panels compared

Geometric morphometric analysis using landmarks to reveal body shape was conducted using Lake Tinnsjøen only, and secondly Lake Tinnsjøen and Lake Tyrivatn in the river drainage to the south of Lake Tinnsjøen. In the latter analysis, the idea was to evaluate the phenotype of the putative ancestral founder that could have colonized Lake Tinnsjøen, and how the Arctic charr in Lake Tyrivatn was morphologically assigned to the FA‐morphs in Lake Tinnsjøen. A Canon EOS 550d camera (Canon lens EFS 18–55 mm and macrolens EFS 60 mm; F20 ISO1600 AV, blitz) was used to photograph (JPEG) fish. Photographs were taken in a Styrofoam box with a permanent standardized light. Fish were placed in natural position with their left side fronting the camera. All fish which had inflated swim bladders were carefully punctuated so that inflation did not affect body shape. After digitalization in TpsUtil 1.53 (Rohlf, 2004a), transforming JPEG to tps‐files, landmarks were scored in TpsDig2 2.16 (Rohlf, 2004b). A set of 30 landmarks (real and semi‐landmarks) were used to capture the body shape of fish, with main focus on the head region (Figure 3a). Similar landmarks have been used in other studies, but there is no consensus regarding the position or number of landmarks to be used. A transparent film with imposed lines helped setting semi‐landmarks. To minimize interindividual scoring bias, all landmarks were set by one person. In MorphoJ 1.06 (Klingenberg, 2011), using the TpsDig2 file, extreme outliers were removed from both datasets after an outlier analysis, followed by a Procrustes fit analysis. A principal component analysis with eigenvalues was conducted for each dataset. As there were still body length effects on shape after PC analyses in MorphoJ (likely due to allometric growth), we corrected for body length using a regression of log centroid size on body shape (PC axes 1–5) in MorphoJ (Klingenberg, 2011) in both datasets, then saving the residuals for further analyses.

To evaluate how concordant body shape was to FA‐morphs in Lake Tinnsjøen, we used a discriminant analysis in JMP 11.2 (SAS institute Inc, 2013) with linear, common covariance using residuals from the five PC axes in MorphoJ. Similarly, we tested morphological resemblance in body shape of the FA‐morphs with their putative ancestral founder from Lake Tyrivatn combining shape data from Lake Tyrivatn in one analysis. Assignment percentages to the categories were recorded for both analyses.

A subset of the catch (see Section 3.2) was used for determining age from otoliths, immersed in 95% EtOH, and read using a microscope (Kristoffersen & Klemetsen, 1991). An unfortunate challenge was encountered as the Arctic charr heads had been stored in unbuffered formalin, which partly prevented age reading in some fish due to unbuffered formalin eating up parts of the otoliths. However, for the age‐determined fish used, we were confident in their age. Further, it was difficult to determine maturity stage in some fish. This situation prevented a thorough life‐history analysis at this stage. Thus, we present age and body weight distributions revealing the youngest sexually mature male and female (also for body weight distributions).

### 2.4 Estimating the degree of reproductive isolation of field‐assigned morphs

A set of 11 microsatellites were amplified and analyzed after procedures in Moccetti et al. (2019) (Appendix S1: Table S2a,b). 3%–6% negative controls per plate and 4% replicate samples were included in the analysis to control cross‐contamination and consistency of genotypes. All negative samples were blank in the fragment analysis, and all replicate samples had matching genotypes. The genotypes were scored in GeneMapper 3.7 (Applied Biosystems) using automatic binning in predefined allelic bins. All genotypes were subsequently verified by visual inspection independently by two persons.

Deviation from Hardy–Weinberg equilibrium (HWE) and linkage disequilibrium (LD (Guo & Thompson, 1992) was estimated using GENEPOP 4.6 (Raymond & Rousset, 1995;Rousset, 2008) implementing an exact test. The presence of LD may lead to erroneous conclusions if loci do not have independent evolutionary histories. Loci exhibiting significant LD should be excluded from analyses. False discovery rate (FDR) corrections (Pike, 2011) were used to test for significant HWE and LD adjusting *p*‐values for multiple tests. The results showed that out of 40 tests of departures from HWE, significant deviations were not found in any loci or populations after FDR correction. Significant LD was discovered between loci SCO204 and SCO218. Thus, locus SCO204 was removed, and a total of 10 loci were used in the subsequent analyses.

GENEPOP 4.6 (Raymond & Rousset, 1995;Rousset, 2008) was used to calculate the number of alleles, expected and observed heterozygosity, and genetic divergence between populations (*F*_ST_) using log‐likelihood‐based exact tests. The software HP‐RARE 1.0 (Kalinowski, 2005) was used to calculate standardized private allelic richness (*A_p_*) and standardized allelic richness (*A_r_*) accounting for differences in sample size. *A_p_* and *A_r_* were calculated with rarefaction using the minimum number of genes in the samples, that is, 28 genes.

The software MICRO‐CHECKER 2.2.3 (Van Oosterhout, Hutchinson, Wills, & Shipley, 2004) was used to check for null alleles, stutter errors, large allele dropout, and size‐independent allelic dropout. Of the ten loci, MICRO‐CHECKER found one locus to exhibit homozygote excess, potentially due to null alleles, being SalF56SFU. Due to the presence of null alleles, the program FREENA (Chapuis & Estoup, 2007;Chapuis et al., 2008) was run to correct for this using the ENA method (Excluding Null Alleles). The FREENA software was run with 5,000 replicates, and corrected *F*_ST_ values were used.

Genetic differentiation (*F*_ST_) was estimated in GENEPOP 4.6 (Raymond & Rousset, 1995;Rousset, 2008) comparing Lake Tinnsjøen and the four outgroup lakes, the four FA‐morphs, and the four outgroup lakes, and among revealed genetically defined morphs (termed GA‐morphs, with a definition of genetic morphs being *q* > 0.7 based on STRUCTURE results; see details below) in Lake Tinnsjøen. *F*_ST_ values are presented with and without the ENA method.

To determine the most likely number of genetic clusters (K), the software STRUCTURE (Pritchard, Stephens, & Donnelly, 2000) was run using 500,000 burn‐in steps and 500,000 Markov chain Monte Carlo (MCMC) repetitions with 10 iterations, considered as a high enough number to reach convergence. STRUCTURE was run a first time with the individuals from Lake Tinnsjøen and the four Norwegian outgroups: Lake Femund, Lake Tyrivatn, Lake Vatnevatnet, and River Leirfossvassdraget. Secondly, a hierarchical approach was performed where the population that deviated the most from the remainder of the populations was removed, and all remaining populations were run a second time. This was repeated until no more clustering was found. The number of genetic clusters was estimated by calculating the logarithmic probability (LnP(K)) and ΔK which is based on changes in K (Evanno, Regnaut, & Goudet, 2005). The most likely number of clusters was determined using STRUCTURE HARVESTER (Earl & Vonholdt, 2012). According to recommendations by Hubisz, Falush, Stephens, and Pritchard (2009), STRUCTURE was also run with the LOCPRIOR function which incorporates geographical sampling locations using default values. Based on K‐clusters results from the STRUCTURE analysis, we assigned different genetic populations or morphs in Lake Tinnsjøen (GA‐morphs). Here, assignment analyses were based on K‐clusters of individuals with *q*‐values of > 0.7 to its own cluster, evaluated as belonging to this population. Individuals with *q*‐values < 0.7 were interpreted as being hybrids of unsure population origin. We further contrasted Lake Tinnsjøen with the four outgroup lakes.

In Lake Tinnsjøen, as for FA‐morphs, association between habitat occurrence and GA‐morphs was tested using χ^2^ statistic in JMP 11.2 (SAS institute Inc, 2013). Further, a discriminant analysis in JMP 11.2 (SAS institute Inc, 2013) was used to test for association between GA‐morphs and FA‐morphs to reveal how concordant these two different morph‐assignment methods were.

As an alternative way to test genetic differentiation, we first conducted a principal component analysis in Genetix 4.05.2 (Belkhir, Borsa, Chikh, Raufaste, & Bonhomme, 2004) based on microsatellite alleles. Then, we tested for differentiation among the lakes for PC1 and PC2 using a nonparametric multiple comparison test (Steel–Dwass all pairs) in JMP 11.2 (SAS institute Inc, 2013). Further, we used the same approach for testing differentiation, now along PC1–3, for four FA‐morphs in Lake Tinnsjøen as described above, by only subsetting Lake Tinnsjøen from the five‐lake dataset.

### 2.5 Phylogeography and the ancestral lineages colonizing Lake Tinnsjøen

DNA was isolated from pectoral fins using the E‐Z96 Tissue DNA Kit (Omega Bio‐tek) following the manufacturer's instructions. Quality and quantity of isolated DNA were assessed using a NanoDrop spectrophotometer and agarose gel electrophoresis. An 851‐base pair fragment of the mitochondrial DNA (mtDNA) cytochrome B (CytB) gene was amplified using a standard primer pair, FishCytB_F (5' ACCACCGTTGTTATTCAACTACAAGAAC 3') and TrucCytB_R (5' CCGACTTCCGGATTACAAGACCG 3') (Sevilla et al., 2007) in 10 µl polymerase chain reactions (PCRs). The reactions consisted of 1 µl 10 x PCR buffer, 0.3 µl 10 µM dNTP, 0.5 µl of each of the 10 µM F and R primers, 5.5 µl ddH_2_O, 0.2 µl Finnzymes DyNAzyme EXT Polymerase, and 2 µl DNA template (0.4–0.8 µg). The cycling profile consisted of an initial 5‐min denaturation step at 94°C, and 32 cycles of 94°C for 30 s, 57°C for 35 s, and 70°C for 1 min, followed by a final 10‐min elongation step at 70°C. The products were treated with ExoZAP^™^ to remove leftover primers and dNTPs, before running the standard BigDye reaction, using the above primer set in 3.5 µM concentrations. The products were cleaned by precipitation, before sequencing them on an ABI 3130XL Automated Genetic Analyzer (Applied Biosystems), using 80‐cm capillaries. All sequences were manually trimmed and verified in Geneious 10 (Biomatters).

For phylogeographical analyses using cytochrome B, the 851‐base pair‐long sequences were aligned in Mega 7.0.26 using default settings (Kumar, Stecher, & Tamura, 2016). Sequences were interpreted mostly based on both forward and reverse readings (but in a few cases, only one sequence direction was readable). A set of 115 Norwegian sequences were obtained where the sample size was 21–22 for the four Lake Tinnsjøen FA‐morphs and 5–9 for the four Norwegian outgroup lakes (Table 5).

For larger scale comparison of phylogeny, highly similar sequences were retrieved using BLAST (https://blast.ncbi.nlm.nih.gov/Blast.cgi) (Appendix S1: Table S2c,d). A cutoff of 200 highly similar sequences were downloaded from BLAST (including various *Salvelinus* taxa), aligned as described above and analyzed with sequences from Lake Tinnsjøen and the four Norwegian outgroup lakes.

The best substitution model for the combined dataset (115 Norwegian and 200 BLAST sequences) was interpreted using online server IQ‐Tree (http://www.iqtree.org/) with 10,000 ultrafast bootstrap iterations (Nguyen, Schmidt, von Haeseler, & Minh, 2015). Here, the best substitution model revealed was TN + F + I (Tamura & Nei, 1993) (Appendix S1: Table S3).

A circular phylogenetic tree using the TN + F + I model was visualized in Treview 1.6.6 (Page, 1996) using all the 88 observed haplotypes from the joint dataset from the 115 Norwegian sequences and 200 BLAST sequences. Earlier, in another tree, we initially used three outgroup taxa to reveal the most ancient haplotypes in the charr sequences: *Salmo trutta* (GenBank accession; LT617532.1), *Oncorhynchus kisutch* (KJ740755.1), and *Coregonus lavaretus* (AJ617501.1). This tree is not shown, but the most ancestral *Salvelinus* sp. sequence revealed from this analysis is presented in the results as the root in the tree.

A map was made (ESRI, 2017) for the joint dataset of the 88 sequences and plotted geographically with regard to a set of selected major clade configurations. Subjective clade definition and selection was done to basically visualize the large‐geographical‐scale patterns of sequences (although alternative clade definitions do exist).

A major large‐scale phylogenetic branch including the Lake Tinnsjøen haplotypes was used for drawing a minimum spanning network in PopART (http://popart.otago.ac.nz) (Bendelt, Forster, & Röhl, 1999), when not considering frequencies of haplotypes. This major clade, which harbored 21 haplotypes, had good statistical support (89%) from the remaining haplotypes and was selected for further resolution, covering a large geographical range. The purpose with this branch selection was to have an in‐depth look at the putative radiation and geographical distribution of the closest genetic relatives to the Lake Tinnsjøen morphs.

For five lakes and FA‐morphs (arranged by mtDNA clades in Lake Tinnsjøen), the number of haplotypes was listed along with genetic diversity estimators in DnaSP v6.11.01 (Rozas et al., 2017). For Lake Tinnsjøen, the association of FA‐morphs or GA‐morphs with the three mtDNA clade frequencies was tested using χ^2^ statistic in JMP 11.2 (SAS institute Inc, 2013).

## 3 RESULTS

### 3.1 Fish catch and field‐assigned morphs

A total of 754 fish were caught in Lake Tinnsjøen, being 457 Arctic charr, 294 brown trout, and 3 perch, and a small number of European minnow (not quantified). For Arctic charr, 63 fish (13.8% of the total catch of Arctic charr) were caught in the pelagial, 105 fish (23.0%) in the littoral, 256 fish (56.0%) in the shallow‐moderate profundal, and 33 fish (7.2%) in the deep profundal (Table 1). For brown trout, 101 fish were caught in the pelagial, 131 in the littoral, and 62 in the profundal. European minnow and perch were only caught in the littoral.

**TABLE 1:** The Arctic charr (*N* = 457) collected in Lake Tinnsjøen in 2013 using different sampling equipment. – denotes equipment not used in that habitat (niche), while a value of 0 denotes equipment used, but no catch in that habitat. The sampling effort was not standardized precluding catch per unit effort

Lake habitat sampled	Habitat code	Depth (m) range	*N* fish total	Benthic nets	Floating nets	Lines	Traps
Pelagial^a^	PEL	0–20	63	–	63	0	–
Littoral^b^	LIT	0–20	105	105	–	0	0
Shallow‐moderate profundal^c^	SDP	20–150	256	173	–	9	74
Deep profundal^d^	ABY	150–350	33	–	–	1	32

^a^ Deposited at < 20 m depth, over depths of > 30 m, and > 50 meters from shore.

^b^ From shore at < 20 m depth.

^c^ From shore at > 20 m and < 150 m depth.

^d^ Deposited at > 150 m depth > 100 m from shore.

In Lake Tinnsjøen, the field‐assigned morphs based on visual appearance (FA‐morphs, *N* = 457) revealed 282 fish (61.7%) of the planktivore morph, 81 fish (17.7%) of the dwarf morph, 62 fish (13.6%) of the piscivore morph, and 32 fish (7.0%) of the abyssal morph (Table 2).

**TABLE 2:** Number and catch percentage of the total catch (*N* = 457) partitioned into field‐assigned morphs (FA‐morphs) in the four lake habitats. The bottom row summarizes the number and catch percentage in the four habitats across the morphs, and the last two columns similarly summarize the catch of the morphs. The abbreviations for the four habitat codes (PEL, LIT, SDP, and ABY) are defined in the footnote of Table 1

FA‐morphs	PEL *N*	%	LIT *N*	%	SDP *N*	%	ABY *N*	%	In morph	%
Planktivore	63	13.8	102	22.3	117	25.6	–	–	282	61.7
Dwarf	–	‐	1	0.2	80	17.5	–	–	81	17.7
Piscivore	–	–	2	0.4	59	12.9	1	0.2	62	13.6
Abyssal	–	–	–	–	–	–	32	7.0	32	7.0
Across morphs	63	13.8	105	23.0	256	56.0	33	7.2	457	

### 3.2 Eco‐morphological and life‐history trait divergence in the charr morphs

In the contingency analysis of FA‐morphs by habitat, the association was significant (*N* = 457, *df* = 9, *R* ^2^ (U) = 0.400, likelihood ratio test; χ^2^ = 387.92, *p* < .0001) (Figure 2b). The planktivore morph was caught in the pelagial (22.3% of the catch within morph), littoral (36.2%), and shallow‐moderate profundal (41.5%), but not in the deep profundal (0%). The dwarf morph was primarily caught in the shallow‐moderate profundal (98.8%) appearing at 20–70 m depths, and only rarely in the littoral (1.2%). The piscivore morph was primarily caught in the shallow‐moderate profundal (95.2%), and rarely in the littoral (3.2%) and deep profundal (1.6%). The abyssal morph was only caught in the deep profundal habitat (100.0%).

With regard to body shape, the first five PC axes were used for analyses capturing a large part of the variation. For Lake Tinnsjøen, PC axes 1–5 explained 45%–4% of the variation in body shape, with a summed variation of 81.5% (PC1 45%, PC2 14%, PC3 13%, PC4 6%, and PC5 4%, respectively). When testing for concordance of body shape and FA‐morphs (Wilks’ lambda 0.20, *F* = 61.25, *df* = 15, *p* < .0001), it was a moderate‐strong concordant assignment ranging from 61.8% (piscivore morph) to 88.5% (abyssal morph) (Table 3, Figure 3b).

**TABLE 3:** Assignment percentage based on discriminant analysis of PC axes 1–5 for body shape comparing the four FA‐morphs in Lake Tinnsjøen. The diagonal values denote “correct” back assignment to original population or morph categories

Comparison	Individuals	Planktivore	Dwarf	Piscivore	Abyssal
Planktivore	266	(88.0)	10.5	1.1	0.4
Dwarf	77	9.1	(74.0)	15.6	1.3
Piscivore	55	–	29.1	(61.8)	9.1
Abyssal	26	–	–	11.5	(88.5)

For life‐history analyses, a subset of 182 out of 457 Arctic charr were successfully used for age analyses (FA‐morphs: planktivore = 85, dwarf = 34, piscivore = 37, abyssal = 26, GA‐morphs; planktivore = 55, dwarf = 30, piscivore = 35 abyssal = 25, hybrids = 10). FA‐morphs and GA‐morphs were visually contrasted regarding weight and age distribution, suggesting large difference among morphs (Figure 3c,d). It seems that the planktivore morph has the lowest age span (1–7 years; mean of 2.9), followed by a roughly equal life span in the dwarf (1–9; 4.8) and abyssal morph (2–11; 5.0). The piscivore morph has the longest life span (4–19; 9.2). There were large differences in weight, where the piscivore morph had the largest size (min–max range of 6–1,816 g; mean of 267 g) followed by the planktivore morph (1–370; 82). The dwarf morph was smaller (2–105; 23), with the abyssal morphs being minute (1–4; 2.2). There was some variation in the youngest sexually mature males (3–6 years) and females (3–6 years) in FA‐ and GA‐morphs. The comparison of FA‐morphs and GA‐morphs broadly gave the same picture with regard to age and weight patterns (Figure 3c,d).

When comparing body shape in Lake Tinnsjøen and Lake Tyrivatn, back assignment (Wilks’ lambda 0.30, *F* = 31.96, *df* = 20, *p* < .0001) showed that Lake Tyrivatn had highest assignment to itself (71.8%), then planktivore morph (18.8%), and lower to dwarf (6.3%) and piscivore (3.1%), and no fish were assigned to abyssal morph (Appendix S1: Table S4).

### 3.3 Estimating the degree of reproductive isolation of field‐assigned morphs

**FIGURE 4:**
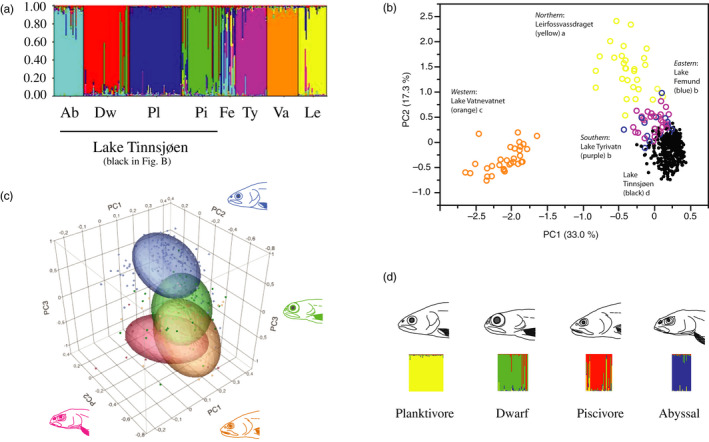
(a) STRUCTURE plot for K = 8 genetic clusters based on the 10 microsatellites for the four Lake Tinnsjøen FA‐morphs and for the four Norwegian outgroup lakes. Abbreviations: Lake Tinnsjøen (Ab = abyssal morph; Dw = dwarf morph; Pl = planktivore morph; Pi = piscivore morph); Fe = Lake Femund; Ty = Lake Tyrivatn; Va = Lake Vatnevatnet; and Le = River Leirfossvassdraget River. (b) PCA plot of microsatellite alleles partitioned into the five lakes studied (different letters denote significant differences on PC1; colors match figure a). (c) Three‐dimensional PCA plot of microsatellite alleles for the four FA‐morphs in Lake Tinnsjøen only (a subset of the four lakes visualized in figure b). The colors in graphs represent heads of the four FA‐morphs on the sides of the graph. (d) STRUCTURE plot for K = 4 based on microsatellites in the FA‐morphs in Lake Tinnsjøen. Note that colors in figure c and d are different and do not correspond to the same morphs across figures

The combined hierarchical STRUCTURE analysis of Lake Tinnsjøen and the four outgroup lakes first showed that there were four separate genetic clusters in Lake Tinnsjøen (Figure 4a,d, Appendix S1: Table S5, hierarchical STRUCTURE plot in Appendix S1: Figure S1). The contingency analysis of FA‐morphs and GA‐morphs was significant (*N* = 344, Df = 12, *R* ^2^ (U) = 0.563, likelihood ratio test; χ^2^ = 453.75 and *p* < .0001) (Table 4). Association ranged from 55.4% (dwarf morph) to 100% (abyssal morph). This implies four genetic populations in Lake Tinnsjøen, concordant with the FA‐morphs. In the combined analysis of Lake Tinnsjøen and outgroup lakes, using principal component on microsatellites, the variation explained along the first two axes was: PC1 (33.0%) and PC2 (17.3%). When contrasting teh FA‐morphs within Lake Tinnsjøen, it was evident that four out of the six comparisons were significantly different for PC1 (*q* = 2.57, alpha = 0.05), and five of six were significantly different for PC2 (Figure 4c). For PC1, the piscivore morph was not different from the abyssal morph, and the planktivore morph was not different from the dwarf. Along PC2, the dwarf morph was not different from the abyssal morph, while for PC3, piscivore and abyssal morph did not differ significantly. In the contingency analysis of habitat‐specific catch by the four revealed GA‐morphs, the association was significant (*N* = 344, *df* = 12, *R* ^2^ (U) = 0.4283, likelihood ratio test; χ^2^ = 302.55 and *p* < .0001), although less than 20% of cells in the tests had expected count < 5 (suggesting x^2^ to be suspect) (Appendix S1: Table S6). The same general pattern emerged as for the FA‐morphs by habitat‐specific catch contingency analysis, implying that the GA‐morphs have different habitat use when compared among themselves.

**TABLE 4:** Association between genetically assigned morphs (GA‐morphs) based on microsatellite‐based STRUCTURE analysis (*q* > 0.70) and the subjectively field‐assigned morphs (FA‐morphs). The group GA–hybrids is fish with a *q*‐value < 0.70 and as such could not be assigned to any specific GA‐morph. Values are percentages within morphs using genetic assignment in GA‐morphs compared to FA‐morphs. The diagonal values denote “correct” back assignment to original population or morph categories

Comparison	Individuals	FA‐planktivore	FA‐dwarf	FA‐piscivore	FA‐abyssal
GA‐planktivore	166	(94.6)	3.0	2.4	–
GA‐dwarf	74	28.4	(55.4)	16.2	–
GA‐piscivore	41	4.9	17.9	(78.0)	–
GA‐abyssal	29	–	–	–	(100.0)
GA‐hybrids	34	14.7	55.9	26.5	2.9

Genetic differentiation was significant among all the four FA‐morphs (also when using ENA correction) showing a range in *F*_ST_ of 0.119–0.199 (*F*_ST_ with ENA correction: 0.119–0.195) (Appendix S1: Table S7). When only considering the “genetically pure” GA‐morphs (*q* > 0.7), *F*_ST_ ranged from 0.088 to 0.212 (*F*_ST_ with ENA correction: 0.087–0.212) (Appendix S1: Table S8).

The combined hierarchical STRUCTURE analysis of Lake Tinnsjøen and the four Norwegian outgroup lakes secondly revealed eight distinct genetic clusters comprising each of the four morphs in Lake Tinnsjøen and each of the four Norwegian outgroup lakes (Figure 4a,d, Appendix S1: Table S5, Appendix S1: Figure S1). The combined analysis of Lake Tinnsjøen and the four Norwegian outgroup lakes using principal components on microsatellites secondly showed that all the lakes were significantly different along PC1 and PC2 (Steel–Dwass method; *q* = 2.72, alpha = 0.05) except for Lake Tyrivatn and Lake Femund that were not significantly differentiated (Figure 4b). Here, Lake Tinnsjøen was most similar to Lake Tyrivatn and Lake Femund. The number of alleles in FA‐morphs and outgroup lakes ranged from 76 (Lake Vatnevatnet) to 143 (planktivore morph), standardized private allele richness from 0.13 (piscivore) to 0.69 (River Leirfossvassdraget), standardized allelic richness from 6.02 (Lake Vatnevatnet) to 8.63 (planktivore morph), *F*_is_ from −0.012 (Lake Tyrivatn and Femund) to 0.118 (River Leirfossvassdraget), heterozygosity from 0.128 (piscivore morph) to 0.820 (Lake Tyrivatn and Femund), and gene diversity from 0.567 (Lake Vatnevatnet) to 0.761 (River Leirfossvassdraget) (Appendix S1: Table S9). Genetic differentiation among the four FA‐morphs and the four outgroup lakes was all significant showing a range in *F*_ST_ of 0.080–0.291 (*F*_ST_ ENA correction: 0.085–0.286) (Appendix S1: Table S7). Here, the planktivore, piscivore, and abyssal morphs were most similar to Lake Femund, while the dwarf morph was most similar to Lake Tyrivatn. When using Lake Tinnsjøen as one group compared with the four outgroup lakes, all were significant, with *F*_ST_ ranging from 0.057 to 0.272 (*F*_ST_ with ENA correction: 0.057–0.269) (Appendix S1: Table S10). Here, Lake Tinnsjøen was most similar to Lake Femund.

### 3.4 Phylogeography and the ancestral lineages colonizing lake Tinnsjøen

A set of 13 haplotypes (*h1–13*) were found in the combined dataset of Lake Tinnsjøen and the four Norwegian outgroup lakes (Table 5). The 13 haplotype sequences obtained in our study are deposited on GenBank (accession numbers: MT276144–MT2761569). Here, 12 of the 13 haplotypes were only found in Lake Tinnsjøen (which lacked *h2*). The four outgroup lakes all had haplotype *h1*, which also occurred in all of the four FA‐morphs, while only one outgroup lake, Lake Vatnevatnet, had an additional haplotype *h2*.

**TABLE 5:** The observed mtDNA haplotypes in Lake Tinnsjøen and in the four Norwegian outgroup lakes. Colors represent three clades where haplotypes group together in the phylogenetic tree (Figure 5b). Summary statistics for genetic variation in the morphs and lakes are also given

Units Haplotype	*N* fish	Planktivore	Dwarf	Piscivore	Abyssal	Tinnsjøen	Leirfoss	Vatnevatnet	Femund	Tyrivatn
Clade I h1	45	1	9	5	2	17	5	8	9	6
h2	1	–	–	–	–	–	–	1	–	–
h10	1	–	–	1	–	1	–	–	–	–
h13	1	–	1	–	–	1	–	–	–	–
Clade II h5	1	–	–	–	1	1	–	–	–	–
h6	23	2	6	1	14	23	–	–	–	–
h7	1	–	1	–	–	1	–	–	–	–
h8	1	1	–	–	–	1	–	–	–	–
h9	1	–	1	–	–	1	–	–	–	–
h11	1	–	–	–	1	1	–	–	–	–
h12	1	1	–	–	–	1	–	–	–	–
Clade III h3	37	16	3	14	4	37	–	–	–	–
h4	1	1	–	–	–	1	–	–	–	–
*N* base pairs	851	851	851	851	851	851	851	851	851	851
*N* sequences	115	22	21	21	22	86	5	9	9	6
*N* haplotypes	13	6	6	4	5	12	1	2	1	1
Variable/singletons	11/8	5/4	5/3	3/1	4/2	10/7	0/0	1/1	0/0	0/0
Parsim. inf. sites	3	1	2	2	2	3	0	0	0	0
Hapl. diversity	0.709	0.476	0.743	0.519	0.576	0.711	0	0.222	0	0
Nucleot. Div. (Pi)	0.00131	0.00086	0.00125	0.00116	0.00078	0.00124	0	0.00026	0	0

**FIGURE 5:**
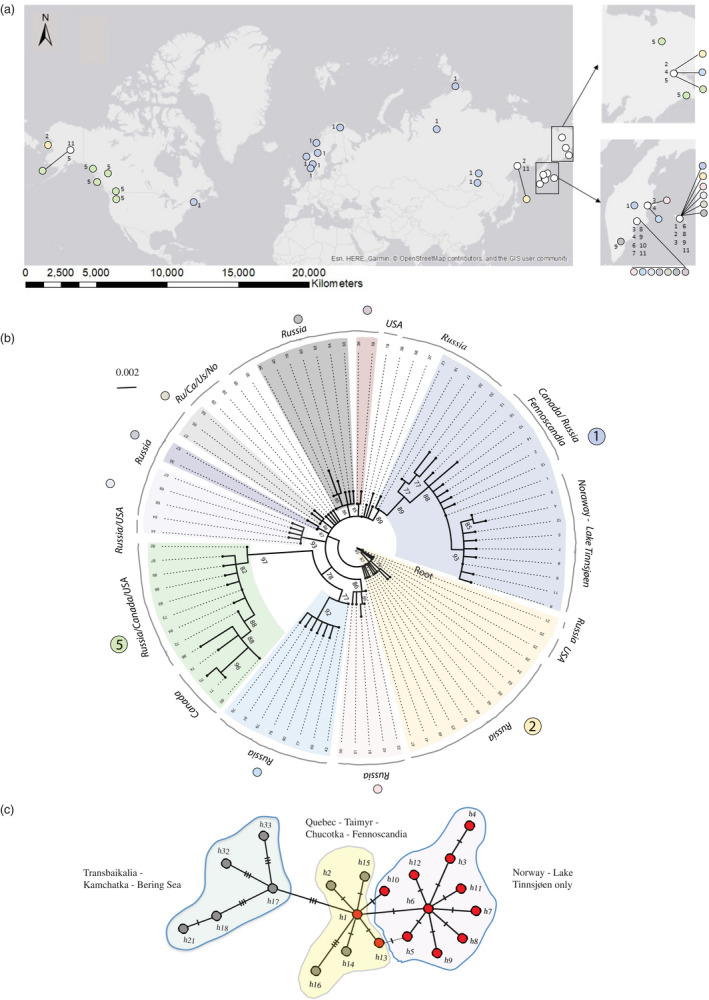
(a) Distribution of 88 mtDNA‐cytochrome B mtDNA haplotypes compared with major clades in different colors according to figure b. White circles denote haplotypes not well supported in figure b. (b) Circular phylogenetic tree of sequences mapped in figure a. Here, a total of 13 Norwegian sequences and 75 haplotypes retrieved from GenBank (using a cutoff of 200 highly similar BLAST sequences) are compared. Here, haplotype 31 was found to be the most ancestral when rooted with three distant salmonid taxa (*Salmo trutta*, *Oncorhynchus kisutch*, and *Coregonus lavaretus*) (tree not shown). Major supported clades have different colors. Main geographical regions are named on the outer circle. (c) A minimum spanning network of haplotypes (not frequencies) in the major light purple clade (#1) comprising Lake Tinnsjøen with geographical areas described. Haplotypes in red were found in Lake Tinnsjøen

From the samples in the larger scale phylogeography (Figure 5a,b), a total of 75 new haplotypes were retrieved from BLAST, comprising 88 haplotypes including the 13 Norwegian haplotypes (Appendix S1: Table S2c,d). Comparing these 75 haplotypes to the ones found in Norway revealed that only *h1* (in five lakes) and *h13* (in one lake) were found outside Lake Tinnsjøen and the four Norwegian outgroups. Lake Tinnsjøen harbored a set of 10 endemic haplotypes (*h3–h12*).

The major branch in Figure 5b (light purple; #1) including Lake Tinnsjøen haplotypes was used for drawing a minimum spanning network, not considering frequencies of haplotypes. This major clade with 21 haplotypes had good statistical support (89%), covering a large geographical range (Figure 5b). Within the light purple clade, a total of 6 haplotypes or subclades were supported with good statistical bootstrap values between 77% and 93%.

In Figure 5b, the phylogeny of the 13 haplotypes in Lake Tinnsjøen reveals moderate‐to‐high bootstrap support for clustering of three “clades”: clade I (*h1, h2, h10, h13*) with bootstrap support of 88%, clade II (*h5–h9, h11, h12*) with bootstrap support of 93%, and clade III (*h3, h4*) with bootstrap support of 85%. Here, clade I consisted of more haplotypes (i.e., *h13–18, h21, h32, h33*) found outside Lake Tinnsjøen and the four Norwegian outgroup lakes. One haplotype link, *h5*–*h13*, had unresolved cluster groupings, where it was interpreted that *h5*, being one mutational step away from *h1*, belonged to clade II rather than to clade I and that *h13* belonged to clade I. The tree topology in Figure 5b and network in Figure 5c support the evaluation. When using FA‐morphs in Lake Tinnsjøen as units, the number of haplotypes ranged from 4 in the piscivore morph to 6 in the dwarf and planktivore morph (Table 5).

In Lake Tinnsjøen, the percentage (Table 5) of the three clades in FA‐morphs showed that the planktivore morph consisted of mostly clade III (77.3%), and less of clade II (18.2%) and clade I (5%). The dwarf had most of clade I (47.6%) and clade II (38.1%) and less of clade III (14.3%). The piscivore morph had most of clade III (66.7%) and less of clade I (28.6%) and clade II (4.8%). Finally, the abyssal morph had most of clade II (72.7%) and less of clade III (18.2%) and clade I (9.1%). The contingency analysis of FA‐morphs and mtDNA clades was significant (*N* = 86, Df = 6, *R* ^2^ (U) = 0.2524, likelihood ratio test; χ^2^ = 46.062 and *p* < .0001) although less than 20% of cells in the tests had expected count < 5 (suggesting x^2^ to be suspect). Here, the planktivore and piscivore morphs had more of clade III, and the abyssal and dwarf morphs had more of clade II than other morphs. The association between GA‐morphs and mtDNA clades was also significant (*N* = 79, *df* = 8, *R* ^2^ (U) = 0.3585, likelihood ratio test; χ^2^ = 60.245 and *p* < .0001) although less than 20% of cells in the tests had expected count < 5 (suggesting x^2^ to be suspect). The same pattern as described above for FA‐morphs appeared.

The genetic diversity (Table 5) of FA‐morphs ranged from a low haplotype diversity of 0.476 (planktivore morph) to a high 0.743 (dwarf morph) with the abyssal morph having a value of 0.576 in Lake Tinnsjøen, and from 0 to 0.222 (highest in Lake Vatnevatnet) in outgroup lakes. In Lake Tinnsjøen combined, the haplotype diversity was found to be 0.711. Similarly for nucleotide diversity, a low value was seen for the abyssal morph (0.00078) and a higher value for the dwarf morph (0.00128), while the four outgroup lakes varied from 0 to 0.00026 (highest in Lake Vatnevatnet). In Lake Tinnsjøen combined, the nucleotide diversity was 0.00124.

## 4 DISCUSSION

We revealed four Arctic charr morphs associated with four habitats in the pelagial (<20 m), littoral (<20 m), shallow‐moderate profundal (20–150 m), and deep profundal (150–350 m) in Lake Tinnsjøen. A novel finding was the abyssal morph in the deep profundal which has not yet been described before in the worldwide Arctic charr species complex. Field assignment from exterior appearance, and laboratory geometric landmark analyses, supported the distinction into four morphs. Life‐history parameters also supported morph separation based on size, age, and maturity patterns. We evaluated that the four morphs were differentiated with regard to habitat use based on catch, and in their life history, suggesting association between phenotypic divergence and catch habitat. This implies adaptive niche proliferation with morphological specialization (due to phenotypic plasticity and/or genomic hardwiring) toward different environmental conditions along the depth–temperature–productivity–pressure gradient in the lake. We found that the four field‐assigned morphs were genetically divergent at microsatellite loci (*F*_ST_: 0.12–0.20), indicating some form of reproductive isolation among morphs. Further, there was a close association between field‐assigned morphs and unbiased genetic analyses (microsatellites) revealing four distinct genetic clusters in the lake, supporting morph differentiation. The genetic differentiation was, partly, also supported by the mtDNA analysis revealing differential clade associations of morphs. We also find it reasonable to postulate that members of one widespread Holarctic mtDNA lineage colonized Lake Tinnsjøen, likely suggesting one single common ancestor that later diversified into the observed four sympatric morphs. Further, the 10 endemic haplotypes found in Lake Tinnsjøen support a mechanism of intralacustrine diversification. Given that this adaptive radiation occurred after the lake became ice‐free (<10,000 years), it represents a rapid diversification in lake niches with associated phenotypic modifications. When considering a 5‐year mean generation time, it corresponds to a maximum of 2,000 generations of evolution. Thus, we found empirical support for evaluating the three main research questions addressed. However, the degree of morphological differentiation, and niche radiation, in Lake Tinnsjøen reveals an extension of specialization into the deep profundal niche. Thus, this highlights an intriguing general question in speciation research of polymorphic fish in lakes: Have we systematically underestimated the degree and rate of adaptive radiation into profundal niches?

### 4.1 What are the main drivers in adaptive radiation of sympatric morphs?

Is there a repeatable pattern in niche use in sympatric morph? Imagine the colonization of a barren lake after the ice age with all lake niches available for utilization. Here, founders will likely utilize the most energetically profitable niche first, depending upon the lake‐specific morphometry with regard to the highest fitness gain in the littoral or pelagial niche. Thus, the starting point for adaptive proliferation may be highly contingent on what niche(s) is actually holding the highest fitness reward among the available lake niches. This will also apply in a situation with presence of another species being a resource competitor or predator. Based on the number of sequence of morphs from monomorphic to four morph systems, it seems that there is a predictable temporal pattern in evolutionary branching associated with niche radiation. Here, the littoral (or pelagial) may be the first niche to be filled, then the pelagial (or littoral), and then the profundal, with a piscivore morph originating putatively due to growth threshold dynamics from one of the units, or evolving independently. Adding upon this complexity, moving away from an assumption of only three discrete niches in a given lake, one can imagine that there could be gradients of predictable fitness along environmental variation such as the depth–temperature–productivity–pressure gradient in Lake Tinnsjøen. Indeed, a study on polymorphic European whitefish (*Coregonus lavaretus*) in the Swizz Alpine Lake Neuchâtel suggested adaptive diversification and buildup of reproductive isolation along ecological gradients when assessing morphs spawning at different time and place (Vonlanthen et al., 2009). Morphological diversification in the north American cisco (*Coregonus* ssp.) species complex has also been related to adaptation by depth in the Canadian Lake Nipigon (Turgeon, Estoup, & Bernatchez, 1999). Ohlberger, Brännström, and Dieckmann (2013) who used an adaptive‐dynamics model, calibrated with empirical data, found support for an evolutionary diversification of the two German Lake Stechlin *Coregonus* sp. morphs likely being driven by selection for physiologically depth‐related optimal temperatures. In the 1.6‐km‐deep Lake Baikal, Russia, one of the oldest freshwater lakes on earth, adaptive radiations have occurred in several taxa such as reflected by the depth gradient and the environmental niche radiation of the freshwater sculpins (*Cottidae*, *Abyssocottidae*, and *Comephoridae*) (Goto, Yokoyama, & Sidelva, 2014). Also, speciation along depth gradients in the ocean is strongly suggested (Ingram, 2011). A study by Chavarie et al. (2018) tested a multitrait depth gradient diversification of morphs in lake trout (*Salvelinus namaycush*) in Bear Lake in Canada, but did not find a strong association in differentiation with depth (but, partly association with genetic structure). In comparison with these studies, it seems reasonable to infer that there is a depth–temperature–productivity–pressure gradient with different fitness rewards reflecting an adaptive landscape whereupon the four Arctic charr morphs within Lake Tinnsjøen can adapt. Such a gradient may not necessarily be discrete with regard to environmental sustainable conditions, but could reflect a continuum, or a holey adaptive landscape (see Gavrilets, 2004). A recent study by Jacobs et al. (2020) revealed the complexity in inferring mechanisms behind origin of replicate Arctic charr morphs. These authors suggested that similar Arctic charr morphs could originate through parallel or nonparallel evolutionary routes as revealed in gene expression being highly similar between independently derived replicates of the same morph. They highlighted that variability in the Arctic charr with regard to predicting phenotypes was contingent on a set of factors such as demographic history, selection response, environmental variation, genomic architecture, and genetic association with specific morphs. Thus, revealing mechanisms in speciation trajectories in the Arctic charr complex is indeed a challenging task.

A novel finding in our study was the appearance of the deep profundal abyssal morph with its distinctive phenotypic features, apparently being adaptations to the cold, dark, and low‐productive high‐pressure environment in deeper parts of the oligotrophic Lake Tinnsjøen. Our finding of the four morphs could reflect a continuum of divergence from surface to the deep profundal environments. This implies large differences in yearly cumulative temperature sum at different depths and productivity, likely strongly affecting life‐history evolution. In shallow Fennoscandian lakes, the littorals seem to have the highest biotic production, followed by the pelagial and profundal (Kahilainen, Lehtonen, & Könönen, 2003). In the 1.6‐km‐deep Lake Baikal, oligochaetes was found from the surface down to maximum depth, comprising up to 70%–90% of biomass and numbers in the bottom fauna (Snimschikova & Akinshina, 1994). In the same lake, biomass of benthos decreased with depth, with an increasing proportion of oligochaetes. In comparison with the Baikal studies, we assume that the biotic prey production for Arctic charr is highest in the pelagial in the deep Lake Tinnsjøen (with small littoral areas) and lower in the benthic–littoral, and the least in the deep profundal. As such, a temperature and food production gradient likely exists in Lake Tinnsjøen from more productive pelagic and littoral areas down to the shallow profundal and deep profundal. Also, as pressure increases by one atmosphere every 10 meters of depth, it should further have marked impacts on adaptations evolved in various traits, being particularly evident in the small abyssal morph with its curved head, upturned mouth, and small eye size. Thus, both abiotic factors and ecological opportunity likely determine the potential of adaptive divergence in deepwater lakes as already implied in studies on Arctic charr in the profundal habitat (Klemetsen, 2010;Knudsen et al., 2006), and in European whitefish (*Coregonus lavaretus*) (Siwertson et al., 2010). In deep lakes such as Tinnsjøen (460 m) and Gander Lake in Canada (288 m; O´Connell, Dempson, & Power, 2005), selective forces for habitat and niche occupation could be even stronger than previously anticipated, selecting for traits that have not been seen in other morphs from other lakes. In Lake Tinnsjøen, the small eyes in the abyssal morph bear apparent similarities with eye reduction seen in cave fishes (e.g., Krishnan & Rohner, 2017). This seems somehow logical given that cave environments often can be described as nutrient‐poor, cold, and harboring few co‐occurring species.

It is pertinent to pose the question whether the Lake Tinnsjøen morphs have originated due to ecological speciation mechanisms. According to the ecological theory of adaptive radiation and ecological speciation (Bernatchez, 2004;Hendry, 2009;Schluter, 2000, 2009), our four morphs do seem to fit well to an ongoing diversification process according to several of the expectations from this theory (see also Hendry, Nosil, & Rieseberg, 2007;Thibert‐Plante & Hendry, 2010a, b, 2011). However, the process of ecological speciation is complex and remains to be tested awaiting ecological niche studies and using higher resolution genetic markers under an evolutionary scenario framework comparing simulated and empirical data. As a crucial and fundamental basis in ecological theory, we would also here, in our newly discovered Lake Tinnsjøen system, expect a niche‐specific fitness trade‐off in adaptations to evolve so that no one phenotype will be optimal in all the available lake niches. Thus, the saying “*Jack of all trades, master of none, but oftentimes better than master of one*” might nicely reflect the early postglacial stages of the ongoing evolutionary dynamics in adaptive radiation of Arctic charr.

### 4.2 Genetic divergence of sympatric morphs in the radiation of Arctic charr

In the Holarctic, the pattern of adaptive diversification in Arctic charr into lake niches seems to be that most lakes hold only one morph (e.g., littoral), fewer lakes have two morphs (e.g., littoral and pelagic), and even fewer lakes have three morphs (e.g., littoral–pelagic and profundal), while only Lake Thingvallavatn, Island, so far has been reported to harbor four morphs (small and large benthic, planktivore, and piscivore). Several studies have compared Arctic charr among lakes with regard to their genetic differentiation (where there may be lakes holding more than one morph of Arctic charr) revealing a microsatellite *F*_ST_ range of 0.003–0.657 when contrasted in Holarctic lakes (Appendix S1: Table S11; including references). The presence of two morphs associated (or not) with genetic clusters has been found in a number of Arctic charr lakes revealing a *F*_ST_ range of 0.006–0.381 (Appendix S1: Table S11, including references). Few lakes harbor three morphs revealing an *F*_ST_ range of 0.017–0.497 (Appendix S1: Table S11; including references). A set of four morphs (small and large dark and small and large pale morphs) have been described from Gander Lake in Canada (O’Connell & Dempson, 2002;Power, O’Connell, & Dempson, 2005). Gomez‐Uchida, Dunphy, and O´Connell, & Ruzzante (2008) tested the dark and pale morphs and found an *F*_ST_ (θ) of 0.136, suggesting two genetic clusters. Currently, it is unknown whether the four morphs in Gander Lake constitute four genetic clusters. The classic textbook example of adaptive radiation in Arctic charr comes from a continental plate rift lava lake, Lake Thingvallavatn, in Iceland. Here, a set of four morphs of Arctic charr has been described: large benthic, small benthic, planktivorous, and piscivorous morphs (Sandlund et al., 1992). Kapralova et al. (2011) studied three of these morphs (small benthic, large benthic, and planktivorous) and found *F*_ST_ (theta) varying between 0 and 0.07. As such, the genetic status of the four Lake Thingvallavatn morphs remains partly unresolved to date with regard to microsatellite differentiation. In our study of the Arctic charr in Lake Tinnsjøen, we estimated *F*_ST_ values between 0.119 and 0.199 among the four morphs, being much more differentiated than the morphs compared in Lake Thingvallavatn. However, the range in genetic differentiation among morphs in Lake Tinnsjøen lies within the range among lakes (*F*_ST_: 0.003–0.657), among two‐morph sympatric systems (*F*_ST_: 0.006–0.381), and within the three‐morph sympatric systems (*F*_ST_: 0.017–0.497). Genetic divergence in mtDNA was also implied among the four sympatric morphs in Lake Tinnsjøen as the morphs were associated with different clade frequencies. With regard to mtDNA divergence of sympatric Arctic charr morphs, much fewer studies exist, mostly at regional or lake‐specific scales to reveal the pattern of divergence (Alekseyev et al., 2009;Salisbury, McCracken, Keefe, Perry, & Ruzzante, 2019;Verspoor, Know, Greer, & Hammar, 2010 **)**. The Arctic charr morphs in Lake Thingvallavatn display low mtDNA differentiation (Danzmann, Ferguson, Skúlason, Snorrason, & Noakes, 1991;Escudero, 2011;Volpe & Ferguson, 1996), and not all morphs are compared, barring a full contrast of the four morphs in Lake Tinnsjøen. Thus, it appears that no direct comparison can be made to relevant studies on Arctic charr considering mtDNA results from Lake Tinnsjøen. However, using the same line of argument as in Alekseyev et al. (2009) and Gordeeva, Alekseyev, Kirillov, Vokin, and Samusenok (2018), one could imply a case of sympatric origin of the four Lake Tinnsjøen morphs as they have endemic haplotypes not yet seen outside the lake. However, that could also reflect limited geographical coverage nearby, or far from, Lake Tinnsjøen. Thus, one should be cautious when interpreting these results.

Genetic divergence (using different markers) among sympatric Arctic charr morphs in lakes throughout the Holarctic varies widely, and we expect them to do so given their different evolutionary histories, genetic load and evolvability, biotic and abiotic environmental conditions, and ecological opportunities to radiate. Indeed, there are systems with one to four morphs in different lakes, but only few studies have addressed nuclear and mtDNA markers at the same time. In Lake Tinnsjøen, we have described four morphs that are different with regard to microsatellites and with regard to frequencies of mtDNA haplotypes. The evolutionary branching in their phylogeny and the high number of endemic haplotypes in Lake Tinnsjøen could support an intralacustrine origin of these morphs. However, the evolutionary scenarios remain to be tested in detail using a set of higher resolution markers. Although the Arctic charr species complex has been studied for a long time, researchers still need to address the important mechanisms underlying origin, presence, and temporal persistence of sympatric morphs. Thus, a multimethod‐based eco‐evo‐devo approach with ecological, morphological, and life‐history studies (Skúlason et al., 2019) and state‐of‐the‐art genomics as performed in Lake Thingvallavatn (Gudbrandsson et al., 2018, 2019) seem to be a good avenue, as well as the methods applied in Jacobs et al. (2020) contrasting two independent replicate lineage radiations of the Arctic charr. Whether or not Lake Tinnsjøen represents a true sympatric speciation process remains to be tested using a combined set of genetic markers to contrast evolutionary scenarios.

### 4.3 Origin and timing of colonization into Lake Tinnsjøen

Identifying whether an ongoing adaptive radiation has a monophyletic origin or results from parallel colonization of several morphs or secondary contact is a daunting task. To provide some initial evidence, we sequenced a mtDNA‐cytochrome B fragment in the four morphs from Lake Tinnsjøen and Arctic charr from four comparative Norwegian populations to the south, west, east, and north of Lake Tinnsjøen. Additionally, we contrasted these results in a Holarctic context, to identify the likely linage(s) colonizing Lake Tinnsjøen. These analyses suggested that the founders of Lake Tinnsjøen carried the *h1* haplotype, widespread in the Holarctic (clade I), subsequently giving rise to clade II (*h5*, *h7*, *h8*, *h9*, *h11*, *h12*) and clade III (*h3*, *h4*), as novel haplotypes within the lake (see also Appendix S2: Information S1 for a phylogenetic discussion). The Norwegian outgroup lakes were all dominated by the haplotype *h1*, and only Lake Vatnevatnet had an additional haplotype *h2*, providing little information about possible routes of colonization into Lake Tinnsjøen. It is therefore relevant to address the glacial geological conditions surrounding the area of Lake Tinnsjøen for evaluating the potential of colonization direction and timing of founder events. The maximum extension of the Eurasian Late Weichselian ice sheet occurred ca 21–23,000 years before present (ybp) (Hughes et al., 2016;Patton et al., 2017). Around 15,000 ybp, the retreating ice margin was close to the Norwegian coast, and the ice stream in the Skagerrak Sea broke up in the Norwegian channel (Longva & Thorsnes, 1997). In southern Telemark county, wherein Lake Tinnsjøen is situated, the ice sheet extended all the way to the coast ca 13,000 ybp (Bergstrøm, 1999). Around 12,000 ybp, the coast was ice‐free (Longva & Thorsnes, 1997). The ice sheet retreated in a northwestern direction. An ice‐recession line southeast of Lake Heddalsvatnet, situated below Lake Tinnsjøen in the same drainage (River Tinne), was dated to 9,700 ybp by Bergstrøm (1999). Further, marine sediment deposits were recorded (www.ngu.no) close to the village of Årlifoss 11 km southeast of Lake Tinnsjøen in River Tinne (see Figure 1b for the position of the upper limit of marine deposits). A sediment core study from Lake Skogstjern in the lower part of the Skiensvassdraget River by Wieckowska‐Lüth, Kirleis, and Doerfler (2017) revealed a lake formation dating at ca 10,500 ybp. The outlet of Lake Tinnsjøen is situated 50 km (estimated current waterway distance) northwest of Lake Heddalsvatnet. Lake Tinnsjøen was glaciated, and we thus assume that it could not have been accessible for fish immigration prior to that period—setting a crude frame for colonization to < 9,700 ybp. We further infer that the fish colonization has proceeded from the southeast through the River Skienselva, or alternatively through any existing nonidentified proglacial lakes situated southeast of Lake Tinnsjøen. This is also logic given the elevation level of the landscape surrounding Lake Tinnsjøen, where colonization along the suggested direction is most likely as the alternative routes imply crossing mountains and elevated slopes. The estimated ice‐flow directions (Figure 1b; Bergstrøm, 1999) support that the Arctic charr colonized Lake Tinnsjøen along the River Skienselva from the coastline and upward. As the Arctic charr can be anadromous and live short periods in the sea (Klemetsen, 2010), and as the Skagerrak area at certain times during deglaciation was carrying a brackish water upper layer (Gyllencreutz, Backman, Jakobsen, Kissel, & Arnold, 2006;Jiang, Björck, & Svensson, 1998), it seems reasonable to infer that the Arctic charr came from the south and colonized Lake Tinnsjøen from the coast.

### 4.4 Conservation biology and management of biodiversity below the species level

Lake Tinnsjøen harbors four significantly genetically differentiated Arctic charr morphs, representing breeding populations with restricted gene flow. These morphs have likely been formed in sympatry within the lake postglacially and may stem from ongoing adaptation to available habitats and resources (niches), as previously implied in yet other Arctic charr systems (Skúlason, Snorrason, & Jónsson, 1999;Snorrason & Skúlason, 2004). If the evolution of these morphs is the result of response to past or prevailing selection pressures, that is, that phenotypic and life‐history differentiation reflects specific adaptation to local conditions, then this may have important management implications. Previously, species have often been considered as the overriding unit in conservation approaches; however, in the last century, also smaller conservation units with the purpose of preserving intraspecific diversity and evolutionary legacies have been developed (Crandall, Bininda‐Emonds, Mace, & Wayne, 2000;Fraser & Bernatchez, 2001;Waples, 1991). Here, one such attempt is evolutionarily significant units (ESUs), defined by Waples (1991), who stated that ESUs are *populations that exhibit substantial reproductive isolation and constitute an important component of the evolutionary legacy of the species.* Moritz (1994) further suggested a more restrictive use of the term ESU and added the criterion that populations should exhibit *reciprocal monophyly for mtDNA haplotypes and significant genetic differentiation at nuclear loci.* Moritz (1994) also suggested the use of a second term, management units (MUs), which excluded the need for reciprocal monophyly, but with the criteria of populations exhibiting significant differentiation at nuclear loci. Crandall et al. (2000) suggested an inclusive ESU concept, as it is mainly focused on historical legacy than on preservation of functional diversity, suggesting the ESU concept should be more holistic including ecology*.* Fraser and Bernatchez (2001) put forward an adaptive evolutionary conservation approach, considering that a context‐based framework should be applied, being more dynamic than strict single criteria or definitions. Mable (2018) discuss the importance of fitness and adaptive potential, species definitions in conservation, type and level of genetic variation, and importance of understanding adaptive processes in the wild for management approaches.

So how do the four Arctic charr morphs in Lake Tinnsjøen fit to concepts issued above? First, the four morphs in Lake Tinnsjøen are not reciprocally monophyletic from each other, but seem to share same haplotypes, although in different frequencies (and with some endemic haplotypes in each morph). The large‐scale phylogeography comparison in our study implies radiation in one of the main branches (clade 1 in Figure 5) where the haplotypes (*h3*–*h13*) in Lake Tinnsjøen are not found elsewhere with the exception of haplotype *h1* which is seen in other populations (i.e., Norway, Finland, Sweden, Russia, Canada). The four morphs are significantly differentiated in their nuclear markers (microsatellites). As such, Lake Tinnsjøen as a whole could be evaluated as comprising one ESU according to Waples (1991), but not according to Moritz (1994). According to Moritz (1994), we would have four MUs in Lake Tinnsjøen, corresponding to the four morphs. In line with Fraser and Bernatchez (2001) and Mable (2018), we support the idea that criteria for evaluation should be more holistic and dynamic considering adaptive diversity and the need to conserve the processes that generate it. The complex Arctic charr system in Lake Tinnsjøen contains extensive genetic variation, but also extensive life‐history variants and phenotypic diversity spanning from the miniscule white abyssal charr to the large piscivore charr. To preserve this degree of local adaptive variation, it is vital to maintain the genetic integrity of the local populations and thereby to conserve the evolutionary potential of the whole lake ecosystem that generated this diversity. Overfishing and other harvest‐related threats are generally not an issue in Lake Tinnsjøen as there is limited use of three of the four morphs. However, other anthropogenic effects such as pollution and in‐lake fish farming could influence water chemistry and enrich the ecosystem with nutrients. Thus, it is imperative to conserve the four morphs together in an undisturbed lake ecosystem. One should prevent negative impacts from, for example, introduction of new species of deepwater‐dwelling piscivore fishes that potentially could decimate the worldwide rare abyssal morph. Hence, the degree of phenotypic and life‐history diversity in Lake Tinnsjøen suggests that the four morphs comprise an important evolutionary legacy of the Arctic charr species complex and offers a rare research window into an ongoing speciation process. As the goal in conservation biology should be to conserve ecological viability and evolutionary processes, capturing the adaptive landscape for evolutionary changes (Fraser & Bernatchez, 2001), the Lake Tinnsjøen ecosystem should merit international biological conservation. In biological conservation, we should not disturb ongoing processes of natural selection, and aim to protect the active units below the species level not only focusing on species conservation. We suggest that the Norwegian management authorities should merit Lake Tinnsjøen special biodiversity protection as it is one of the most divergent Arctic charr systems seen worldwide.

## CONFLICT OF INTEREST

The authors declare no conflict of interest.

## AUTHORS’ CONTRIBUTION

K.P. and K.Ø. conceived/designed the study (equal project leaders). All authors contributed to the field sampling (with exception of A‐M.P.T.). Basic genetic work in the laboratory was conducted by M.H.H, M.H., and K.P. Analyses of fish morphology were done by M.H.H. and M.H. Genetic analyses were done by K.P., M.H., M.H.H., A.‐M.P.T., and K.Ø. The main body of the manuscript was written by K.Ø., K.P., and M.H.H. with significant contributions from all co‐authors. All authors read and approved the final manuscript.

## FISHING LICENSE

Fish were sampled after initial consent from local authorities at Tinn County Administration giving us permission to fish in Lake Tinnsjøen after consent was approved also by the local landowners. The local landowners gave us the oral permission to fish on their land. No other permit or ethics approval is needed in Lake Tinnsjøen in order to sample Arctic charr when the main purpose is to use Arctic charr for scientific studies.

## Supporting information

Appendix S1

Table S2b

Table S2c

Table S2d

Appendix S2

## Data Availability

Parts of the data used in this study are available as online Appendix in the electronic version of the article. The reason why not all data have been freely distributed is the current unknown status of the abyssal morph described in our survey. As such, conservation authorities should evaluate the taxonomic status and conservation need of the morph before information on specific sampling locations and catches may be released. This is a logic precautionary conservation biological approach as the observed new abyssal morph in the deep profundal habitat (150–350 m) may have a small/vulnerable population size. Further, as this morph is not found elsewhere in the world, it merits the highest conservation status possible. Lake Tinnsjøen represents a unique window into speciation for scientists.
