# Supplementary material for: “And if you gaze long into an abyss, the abyss gazes also into thee”: four morphs of Arctic charr adapting to a depth gradient in Lake Tinnsjøen
Source: Evol Appl. 2020 Jun 26;13(6):1240–61. doi: 10.1111/eva.12983 (PMC7359846; doi:10.1111/eva.12983)
Supplement: Supplementary file 1 — Appendix S1 [file EVA-13-1240-s001.docx]

**ADDITIONAL FILE INFORMATION**

**TABLE S1** Summary table for number of arctic char used in analyses. * Evaluated after microsatellite analyses.

| Lake/river/  source | FA-morph | Sampled - N | Body shape -N | MtDNA - N | Microsatellites - N | Weigth FA | Weigth GA | Age FA | Age GA |
| --- | --- | --- | --- | --- | --- | --- | --- | --- | --- |
| Tinnsjøen | Planktivore | 282 | 266 | 22 | 185 | 282 | 166 | 85 | 55 |
|  | Dwarf | 81 | 77 | 20 | 73 | 81 | 74 | 34 | 30 |
|  | Piscivore | 62 | 55 | 22 | 57 | 62 | 41 | 37 | 35 |
|  | Abyssal | 32 | 26 | 22 | 30 | 32 | 29 | 26 | 25 |
|  | Hybrids * | - | - | - | - | - | 34 | - | 10 |
| Tyrivatn | - | 32 | 32 | 6 | 32 | - | - | - | - |
| Vatnevatnet | - | 64 | - | 9 | 32 | - | - | - | - |
| Leirfoss | - | 29 | - | 5 | 29 | - | - | - | - |
| Femund | - | 14 | - | 9 | 14 | - | - | - | - |
| Genbank | - | - | - | 77 | - | - | - | - | - |
| Sum |  | 567 | 456 | 192 | 452 | 457 | 344 | 182 | 155 |

**TABLE S2a** The basic description of the 10 microsatellites that was used in arctic char in Lake Tinnsjøen and in the four Norwegian outgroups.

| **Locus** | **Range base pair (bp) in our study** | **Sequence repeat motif (reference in last column)** | **Initially described motif** | **GenBank accesession number** | **Reference for microsatellite markers** |
| --- | --- | --- | --- | --- | --- |
| OMM1105 ^1^ | 112-194 | (AGAC)_23_ (GATA)_16_ | Not defined | AF352768 | Rexroad et al., (2001) |
| SalF56SFU ^2^ | 183-211 | (TG)_25_ | Dinucleotide | AF537307 | McGowan et al., (2004) |
| SalP61SFU ^3^ | 108-164 | (CA)_18_ | Dinucleotide | AF537312 | McGowan et al., (2004) |
| Sco204 ^*^ | not given | (TCTA)_29_ | Tetranucleotide | AY88873 | DeHaan & Arden, (2005) |
| Sco212 ^4^ | 226-382 | (ATCT) _18_ | Tetranucleotide | AY88881 | DeHaan & Arden, (2005) |
| Sco218 ^5^ | 161-245 | (GATA)_31_ | Tetranucleotide | AY88887 | DeHaan & Arden, (2005) |
| Sco220 ^6^ | 239-367 | (ATAG)_5_(ATAC)_2_(ATAG)_15_- | Tetranucleotide | AY88889 | DeHaan & Arden, (2005) |
| SMM17 ^7^ | 105-135 | (CA)_29_ | Dinucleotide | AY327127 | Crane et al., (2004) |
| SMM22 ^8^ | 155-271 | (TAGA)_19_ | Tetranucleotide | AY327129 | Crane et al., (2004) |
| SalJ81SFU ^9^ | 101-159 | (GT)_33_ | Dinucleotide | AF537304 | McGowan et al., (2004) |
| Sco215 ^10^ | 275-283 | (GAAA)_16_(GA)_6_- | Tetranucleotide | AY88884 | DeHaan & Arden, (2005) |

References:

Crane, P. A., Lewis. C. J., Kretschmer, E. J., Miller, S. J., Spearman, W. J., DeCicco, A. L., Lisac, M. J., & Wenburg, J.K. (2004). Characterization and inheritance of seven microsatellite loci from Dolly Varden, *Salvelinus malma*, and cross-species amplification in Arctic char, *S. alpinus*. *Conservation Genetics*, 5, 737-741.

DeHaan, P. W., & Ardren, W. R. (2005). Characterization of 20 highly variable tetranucleotide microsatellite loci for bull trout (*Salvelinus confluentus*) and cross amplification in other *Salvelinus* species. *Molecular Ecology Notes*, 5, 582-585.

McGowan, C. R., Davidson, E. A., Woram, R. A., Danzmann, R. G., Ferguson, M. M., & Davidson, W. S. (2004). Ten polymorphic microsatellite markers from Arctic charr (*Salvelinus alpinus*): linkage analysis and amplification in other salmonids. *Animal Genetics*, 35, 462-504.

Rexroad III, C. E. , Coleman, R. L., Herschberger, W. K., & Killefer, J. (2012). Rapid communication: Thirty-eight polymorphic microsatellite markers for mapping in rainbow trout. *Journal of Animal Science*, 80, 541-542.

* This locus was not used in our analysis as it was in LD with another locus, thus no range in basepairs have been provided.

**TABLE S2b.** The raw data of 10 microsatellites used in arctic char in Lake Tinnsjøen and 4 Norwegian outgroups. This is a separate Excel table.

**TABLE S2c** The accession numbers and information about the 88 Cytochrome B - mtDNA haplotypes compared in Holarctic *Salvelinus* sp. including Lake Tinnsjøen and the four Norwegian outgroups and similar sequences retrieved from GenBank. This is a separate Excel table.

**TABLE S2d** The 88 Cytochrome B - mtDNA haplotypes compared in Holarctic *Salvelinus* sp. including Lake Tinnsjøen and the four Norwegian outgroups and similar sequences retrieved from GenBank. This is a separate FASTA file.

**TABLE S3** The best substitution model selection in IQ-Tree (http://www.iqtree.org/)

(Hoang et al., 2018; Kalyaanamoorthy et al., 2017; Nguyen et al., 2015) with the best five models

for the combined dataset of 88 haplotypes (13 Norwegian haplotypes and the 75 haplotypes found in

Genbank). The best model selected was based on BIC was TN + F + I.

| Model | LogL | AIC | w-AIC | AICcc | w-AICc | BIC | w-BIC |
| --- | --- | --- | --- | --- | --- | --- | --- |
| TN+F+I | -1986.5 | 4331.1 | +0.1323 | 4427.1 | +0.2382 | 5180.7 | +0.5160 |
| TN+F+G4 | -1987.1 | 4332.1 | +0.0765 | 4428.2 | +0.1377 | 5181.8 | +0.2982 |
| TN+F+I+G4 | -1985.3 | 4330.7 | +0.1599 | 4427.9 | +0.1566 | 5185.0 | +0.0581 |
| TIM2+F+I | -1985.8 | 4331.7 | +0.0969 | 4428.9 | +0.0949 | 5186.0 | -0.0352 |
| TIM3+F+I | -1986.3 | 4332.7 | +0.0589 | 4429.9 | +0.0577 | 5187.0 | -0.0214 |

References:

Hoang, D. T., Chernomor, O., von Haseler, A., Minh, B. Q., & Vinh, L. S. (2018). UFBoot2: Improving the ultrafast boostrap approximation. *Molecular Biology and Evolution*, 35, 518-522.

Kalyaanamoorthy, S., Minh, B. Q., Wong, T. K. F., von Haeseler, A., & Jermiin, L. S. (2017). ModelFinder: Fast model selection for accurate phylogenetic estimates. *Nature Methods*, 14, 587-589.

Nguyen, L.-T., Schmidt, H. A., von Haeseler, A., & Minh, B. Q. (2015). IQ-TREE: A fast and effective stochastic algorithm for estimating maximum likelihood phylogenies. *Molecular Biology and Evolution*, 32, 268-274. doi: 10.1093/molbev/msu300

**TABLE S4** The back assignment percentage based on discriminant analysis of PC-axis 1-5 for

body shape in four FA-morphs in Lake Tinnsjøen compared a putative ancestor in Lake Tyrivatn.

The diagonal (values) denote “correct” back assignment to original population or morph categories.

| Comparison | N | Lake Tyrivatn | Planktivore | Dwarf | Piscivore | Abyssal |
| --- | --- | --- | --- | --- | --- | --- |
| Lake Tyrivatn | 32 | (71.8) | 18.8 | 6.3 | 3.1 | - |
| Planktivore | 266 | 14.7 | (70.3) | 13.5 | 1.5 | - |
| Dwarf | 78 | 15.4 | 16.7 | (46.1) | 15.4 | 6.4 |
| Piscivore | 55 | 3.6 | 3.6 | 23.6 | (58.3) | 10.9 |
| Abyssal | 32 | - | - | 3.8 | 15.4 | (80.8) |

**TABLE S5** Output of hierarchical microsatellite STRUCTURE analyses in STRUCTURE-HARVESTER

evaluations regarding the most likely K clusters in Lake Tinnsjøen and the four Norwegian outgroup lakes.

The most likely number of K clusters were interpreted to be K=8 based on these hierarchical analyses.

|  | K | LnP(K) | Stdev LnP(K) | Ln'(K) | \|Ln''(K)\| | DeltaK |
| --- | --- | --- | --- | --- | --- | --- |
| Run 1 | 5 | -16833.62 | 2.25 | 425.38 | 719.34 | 319.24 |
| Run 2 | 6 | -15537.47 | 106.88 | 481.33 | 926.71 | 8.67 |
| Run 3 | 4 | -14525.71 | 3.87 | 471.50 | 360.12 | 92.94 |
| Run 4 | 5 | -16833.62 | 2.25 | 425.38 | 719.34 | 319.24 |
| Run 5 | 4 | -12521.01 | 85.83 | 118.74 | NA | NA |

**TABLE S6** The association between genetically assigned morphs (GA-morphs) based

on STRUCTURE where “pure” morphs have q<0.7 and hybrids have q>0.7 and their catch in

the four lake habitats. The abbrevations for the four habitat codes (PEL, LIT, SDP, ABY) are

defined in the footnote of Table 1. Values are percentages within morphs (rows) while the bottom row

summarize overall percentage of catch in the four lake habitats. The last column summarize

the overall percentage in the field catches with regard to relative percentage of GA-morphs.

| GA-morphs | N | PEL | LIT | SDP | ABY | % of catch |
| --- | --- | --- | --- | --- | --- | --- |
| Planktivore | 166 | 19.3 | 33.1 | 47.6 | - | 48.3 |
| Dwarf | 74 | - | 5.4 | 93.2 | 1.4 | 21.5 |
| Piscivore | 41 | - | - | 100.0 | - | 11.9 |
| Abyssal | 29 | - | - | - | 100.0 | 8.4 |
| Hybrids | 34 | - | 2.9 | 94.1 | 2.9 | 9.9 |
| % in habitats | 344 | 9.3 | 17.4 | 64.3 | 9.0 | 100.0 |

**TABLE S7** Microsatellite F_ST_ values for FA-morphs in Lake Tinnsjøen and outgroup lakes. The lower

diagonal is conventional methods and upper diagonal is ENA corrections. All comparisons are significant.

| Comparison | Abyssal | Dwarf | Planktivore | Piscivore | Femund | Tyrivatn | Vatnevatnet | Leirfoss |
| --- | --- | --- | --- | --- | --- | --- | --- | --- |
| Abyssal |  | 0.130 | 0.144 | 0.158 | 0.117 | 0.174 | 0.251 | 0.170 |
| Dwarf | 0.136 |  | 0.119 | 0.132 | 0.127 | 0.107 | 0.265 | 0.179 |
| Planktivore | 0.149 | 0.119 |  | 0.195 | 0.085 | 0.147 | 0.257 | 0.140 |
| Piscivore | 0.160 | 0.133 | 0.196 |  | 0.165 | 0.208 | 0.286 | 0.217 |
| Femund | 0.119 | 0.128 | 0.080 | 0.168 |  | 0.117 | 0.247 | 0.109 |
| Tyrivatn | 0.179 | 0.109 | 0.147 | 0.210 | 0.117 |  | 0.269 | 0.159 |
| Vatnevatnet | 0.259 | 0.268 | 0.261 | 0.291 | 0.251 | 0.272 |  | 0.233 |
| Leirfoss | 0.175 | 0.183 | 0.142 | 0.218 | 0.108 | 0.160 | 0.234 |  |

**TABLE S8** Microsatellite based F_ST_ values for the “genetically pure” (i.e.

q>0.7) GA-morphs in Lake Tinnsjøen. Lower diagonal is conventional method

and upper diagonal is ENA correction. All the comparisons are significant.

| Comparison | Planktivore | Dwarf | Piscivore | Abyssal |
| --- | --- | --- | --- | --- |
| Planktivore |  | 0.087 | 0.212 | 0.130 |
| Dwarf | 0.088 |  | 0.185 | 0.126 |
| Piscivore | 0.212 | 0.185 |  | 0.201 |
| Abyssal | 0.135 | 0.131 | 0.201 |  |

**TABLE S9** Genetic diversity measures for microsatellites summarized for the four Lake Tinnsjøen morphs and four outgroup lakes.

| Morph/population | Number of alleles | Standardized private allele richness | Standardized allelic richness | F_IS_ | Heterozygosity | Gene diversity |
| --- | --- | --- | --- | --- | --- | --- |
| Lake Tinnsjøen | 163 | 0.29 | 8.09 | 0.020 | 0.560 | 0.750 |
| *Planktivore* | 185 | 0.38 | 8.63 | -0.006 | 0.802 | 0.690 |
| *Dwarf* | 73 | 0.22 | 8.23 | 0.020 | 0.674 | 0.677 |
| *Piscivore* | 57 | 0.13 | 7.46 | 0.035 | 0.128 | 0.602 |
| *Abyssal* | 30 | 0.41 | 8.03 | 0.048 | 0.653 | 0.683 |
| Lake Tyrivatn | 32 | 0.52 | 7.56 | -0.012 | 0.820 | 0.683 |
| Lake Vatnevatnet | 32 | 0.68 | 6.02 | 0.024 | 0.160 | 0.567 |
| River Leirfoss | 29 | 0.69 | 7.53 | 0.118 | 0.178 | 0.761 |
| Lake Femund | 14 | 0.52 | 7.56 | -0.012 | 0.820 | 0.683 |

**TABLE S10** The microsatellite F_ST_ values for the five lakes studied when

combining four FA-morphs in Lake Tinnsjøen. Lower diagonal is conventional

method and upper diagonal is ENA corrections. All comparisons are significant.

| Comparison | Tinnsjøen | Femund | Tyrivatn | Vatnevatnet | Leirfoss |
| --- | --- | --- | --- | --- | --- |
| Tinnsjøen |  | 0.057 | 0.101 | 0.199 | 0.113 |
| Femund | 0.057 |  | 0.117 | 0.247 | 0.109 |
| Tyrivatn | 0.100 | 0.117 |  | 0.269 | 0.159 |
| Vatnevatnet | 0.203 | 0.251 | 0.272 |  | 0.233 |
| Leirfoss | 0.117 | 0.108 | 0.160 | 0.234 |  |

**TABLE S11** Summary table for genetic differentiation from microsatellites among Arctic charr lakes and morphs in the Holarctic. Different number and types of microsatellite loci have been used in the different studies. References are given below the table and do not appear in the main text. In the among-lakes comparisons it is unkown if lakes harbour more than one morph based on what is presented in the references.

| **Comparison** | **F_ST_ range** | **Note** | **References** |
| --- | --- | --- | --- |
| Individual study reports:  “among lakes” | 0.095 - 0.283  0.009 - 0.124  0.003 - 0.171  0.087 - 0.657  0.010 - 0.470  0.017 - 0.283  0.022 - 0.627  0.122 - 0.437  0.056 - 0.381 | Overall F_ST_ (Ɵ) of 0.245  L. Tazimina Lake vs others F_ST_ 0.053 - 0.178  Overall F_ST_ of 0.360 (wild populations)  Overall F_ST_ of 0.260  Overall F_ST_ of 0.267 | Adams et al., (2008)  Bernatchez et al., (1998)  Bernatchez et al., (2002)  Brunner, (1998)  Kapralova et al., (2011)  May-McNally et al., (2015)  Primmer, (1999)  Shikano et al., (2015)  Wilson et al., (2004) |
| Range “among lakes”: | 0.003 - 0.657 |  | Based on the 9 studies above. |
| Individual study reports:  “between two morphs” | 0.168 - 0.180  0.030 - 0.299  0.041  0.032  0.056 - 0.381  0.025 - 0.060  0.008 - 0.321  0.188  0.006  0.006 - 0.014  0.121 |  | Adams et al., (2008)  Gordeeva et al., (2015)  Moccetti et al., (2019)  Westgaard et al., (2004)  Wilson et al., (2004)  Kapralova et al., (2008)  Gíslason et al., (1999)  Power et al., (2009)  Arbour et al., (2011)  Corrigan et al., (2011)  Præbel et al., (2016) |
| Range “between two morphs”: | 0.006 - 0.381 |  | Based on the 11 studies above. |
| Individual study reports:  “Among three morphs” | 0.168 - 0.299  0.033 - 0.059  0.168 - 0.497  0.017 - 0.092  0.042 - 0.134 |  | Alekseyev et al., (2014)  Gíslason et al., (1999)  Gordeeva et al., (2015)  May-McNally et al., (2015)  Moccetti et al., (2019) |
| Range “among three morphs”: | 0.017 - 0.497 |  | Based on the 5 studies above. |
| Range “among four morphs”: | 0.130 - 0.195 |  | Based on Østbye et al., (2020) |

References:

Adams, C. E., Wilson, A. J., & Ferguson, M. M. (2008). Parallel divergence of sympatric genetic and body size forms of Arctic charr, *Salvelinus alpinus*, from two Scottish lakes. *Biological Journal of the Linnean Society*, 95, 748­-757. doi.org/10.1111/j.1095-8312.2008.01066.x

Alekseyev, S., Gordeeva, N., Matveev, A. N., Samusenok, V. P., Vokin, A. I., & Yur`ev, A. L. (2014). Three sympatric forms of Arctic charr *Salvelinus alpinus* complex (Salmoniformes, Salmonidae) from Lake Kamkanda, Northern Transbaikalia. *Journal of Ichthyology*, 54, 384-408. doi: 10.1134/S0032945214040018

Arbour J. H., Hardie, D. C., & Hutchings, J. A. (2011). Morphometric and genetic analyses of two sympatric morphs of the Arctic char (*Salvelinus alpinus*) in the Canadian high arctic. *Canadian Journal of Zoology*, 89, 19-30. doi.org/10.1139/Z10-100

Bernatchez, L., Dempson, J. B., & Martin, S. (1998). Microsatellite gene diversity analysis in anadromous Arctic char, *Salvelinus alpinus*, from Labrador, Canada. *Canadian Journal of Fisheries and Aquatic Sciences*, 55,1264-1272. doi.org/10.1139/f97-325

Bernatchez, L., Rhydderch, J. G., & Kircheis, F. W. (2002). Microsatellite diversity analysis in landlocked Arctic char from Maine. *Transactions of the American Fishery Society*,131, 1106-1118.

Brunner, P. C., Douglas, M. R., & Bernatchez, L. (1998). Microsatellite and mitochondrial DNA assessment of population structure and stocking

effect in Artic char *Salvelinus alpinus* (Teleostei: Salmonidae) from central alpine lakes. *Molecular Ecology*, 7, 209-223. doi.org/10.1046/j.1365-294x.1998.00341.x

Corrigan, L. J., Lucas, M. C., Winfield, I. J., & Hoelzel, A. R. (2011). Environmental factors associated with genetic and phenotypic divergence among sympatric populations of Arctic charr (*Salvelinus alpinus*). J*ournal of Evolutionary Biology*, 24, 1906-1917. doi: 10.1111/j.1420-9101.2011.02327.x

Gíslason, D., Ferguson, M. M., Skulason, S., & Snorrason, S. S. (1999). Rapid and coupled phenotypic and genetic divergence in Icelandic Arctic charr (*Salvelinus alpinus*). *Canadian Journal of Fisheries and Aquatic Sciences*, 56, 2229-2234. doi.org/10.1139/f99-245

Gordeeva, N. V., Alekseyev, S. S., Matveev, A. N., & Samusenok, V. P. (2015). Parallel evolutionary divergence in Arctic charr *Salvelinus alpinus* complex from Transbaikalia: variation in differentiation degree and segregation of genetic diversity among sympatric forms. *Canadian Journal of Fisheries and Aquatic Sciences*, 72, 96-115. doi.org/10.1139/cjfas-2014-0014

Kapralova, K. H. (2008). Genetic population structure of small benthivorous and planctivorous Artic charr (*Salvelinus alpinus*(L.)) in Thingvallavatn, Iceland. Department of biology. Thesis.

Kapralova, K. H., Morrissey, M. B., Kristjansson, B. K., Olafsdottir, G. A., Snorrason, S. S., & Ferguson, M. M. (2011). Evolution of adaptive diversity and genetic connectivity in arctic charr (*Salvelinus alpinus*) in Iceland. *Heredity*, 106, 472-487. doi: 10.1038/hdy.2010.161

May‐McNally, S. L., Quinn, T. P., Woods, P. J., & Taylor, E. B. (2015). Evidence for genetic distinction among sympatric ecotypes of Arctic char (*Salvelinus alpinus*) in south‐western Alaskan lakes. *Ecology of Freshwater Fish*, 24, 562-574.  doi.org/10.1111/eff.12169

Moccetti, P., Siwertsson, A., Kjær, R., Amundsen, P.-A., Præbel, K., Tamayo AMP, Power, M., & Knudsen, R. (2019). Contrasting patterns in trophic niche evolution of polymorphic Arctic charr populations in two subarctic Norwegian lakes. *Hydrobiologia*, 840, 281. doi.org/10.1007/s10750-019-3969-9

Power, M., Power, G., Reist, J. D., & Bajno, R. (2009). Ecological and genetic differentiation among the Arctic charr of Lake Aigneau, Northern Quebec. *Ecology of Freshwater Fish*, 18, 445-60. doi.org/10.1111/j.1600-0633.2009.00362.x

Primmer, C. R., Aho, T., Estoup, A., & Cornuett, J. - M. (1999). Microsatellite analysis of hatchery stocks and natural populations of Arctic charr, *Salvelinus alpinus*, from the Nordic region: implications for conservation. *Heriditas*, 130, 277-289. doi.org/10.1111/j.1601 5223.1999.00277.x

Præbel, K., Couton, M., Knudsen, R., & Amundsen, P. - A. (2016). Genetic consequences of allopatric and sympatric divergence in Arctic charr

(*Salvelinus alpinus* (L.)) from Fjellfrøsvatn as inferred by microsatellite markers. *Hydrobiologia*, 783, 257-267. doi.org/10.1007/s10750-016-2648-3

Shikano, T., Järvinen, A., Marjamäki, P., Kahilainen, KK, & Merilä, J. (2015). Genetic variability and structuring of Arctic charr (*Salvelinus alpinus*) populations in Northern Fennoscandia. *Plos One*,10, e0140344. doi.org/10.1371/journal.pone.0140344

Westgaard, J. L., Klemetsen, A., & Knudsen, R. (2004). Genetic differences between two sympatric morphs of Arctic charr confirmed by microsatellite DNA. *Journal of Fish Biology*, 65,1185-1191. doi.org/10.1111/j.0022-1112.2004.00524.x

Wilson, A. J., Gíslason, D., Skúlason, S., Snorrason, S., Adams, C. E., Alexander, G., Danzmann, R. G., & Ferguson, M. M. (2004). Population genetic structure of Arctic Charr, *Salvelinus alpinus* from northwest Europe on large and small scales. *Molecular Ecology,* 13,1129-1142 doi: 10.1111/j.1365-294X.2004.02149.x

**FIGURE S1** Hierarchical STRUCTURE plot using the four morphs in Lake Tinnsjøen and the four Norwegian outgroup lakes. Codes; TY (Lake Tyrivatn), FE (Lake Femund), NO (Planktivore morph Lake Tinnsjøen), DD (Absyssal morph Lake Tinnsjøen), DS (Dwarf morph Lake Tinnsjøen), PI (Piscivore morph Lake Tinnsjøen), VA (Lake Vatnevatnet), STO (River Leirfossvassdraget).

**
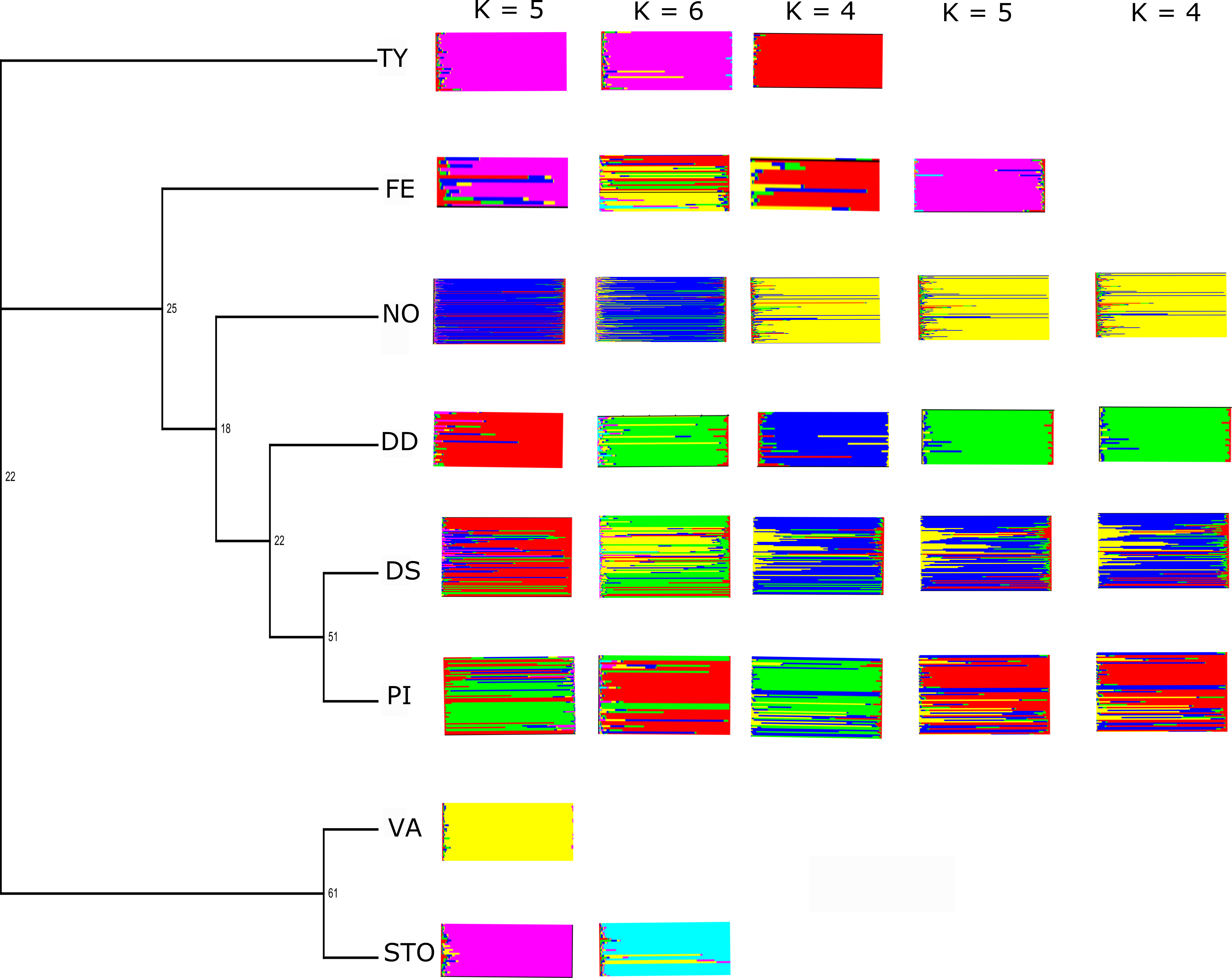
**
