# Supplementary material for: “And if you gaze long into an abyss, the abyss gazes also into thee”: four morphs of Arctic charr adapting to a depth gradient in Lake Tinnsjøen
Source: Evol Appl. 2020 Jun 26;13(6):1240–61. doi: 10.1111/eva.12983 (PMC7359846; doi:10.1111/eva.12983)
Supplement: Supplementary file 5 — Appendix S2 [file EVA-13-1240-s005.docx]

**ADDITIONAL FILE: INFORMATION S1** Information related to phylogenetic discussion.

**4.5** | **Holarctic phylogeography and lineages likely colonizing Lake Tinnsjøen**

We have screened a moderate number of each of the four morphs in Lake Tinnsjøen and only few fish in the four comparative Norwegian populations to the South, West, East and North of Lake Tinnsjøen for one mtDNA Cytochrome B fragment. In our study, these lakes hold Arctic charr as we would phenotypically recognize them in Norway, with perhaps the exception of the abyssal morph which is morphologically distinct. We used our sequences to obtain similar sequences from 18 named species of *Salvelinus* from GenBank to assess Holarctic patterns in haplotypes, lineages, clades and taxa distributions (Table 3, Additional file: Table S2c,d). The initial description of taxa in GenBank sequences comprised; *Salvelinus* spp, *Salvelinus albus*, *S. andrashevi*, *S. alpinus*, *S. alpinus alpinus*, *S. alpinus erythrinus*, *S. boganidae*, *S. confluentus*, *S. elgyticus*, *S. krogiusae*, *S. kronocius*, *S. kuznetzovi*, *S. malma*, *S. malma malma*, *S. malma lordi*, *S. neiva*, *S. oquassa*, *S. schmidti*, and *S. taranetzi*. Here, we used the sequences from GenBank regardless of species denomination in the initial deposition or publications. This strategy was chosen as the species designation in the *Salvelinus* complex is challenging due to the traditional use of morphological species criteria with variable consensus. When considering all 88 haplotypes together, *S. alpinus* was described with haplotypes; *h1-h17*, and *h82*, while remaining taxa belonged to other taxa than *S. alpinus*. Here, Norway, Sweden, Finland, Canada, USA, and Russia report haplotypes of *S. alpinus*. Our Holarctic CytB phylogeny, and phylogeographic patterns, reveals a much deeper divergence than only considering the single Arctic charr *S. alpinus* taxa. As mtDNA is maternally inherited it only partly reveals the evolutionary history of the *S. alpinus* species complex. In general, one should be cautious inferring mtDNA based phylogeography as we assume selective neutrality, which may not always be the case (e.g. Consuegra et al., 2018).

Our main purpose of the large scale comparison of CytB sequences (using different described charr (*Salvelinus* spp.) taxa) was to visualize, and polarize, the closest relatives in the major branch that also contained the Arctic charr in Lake Tinnsjøen. With that in mind, we focused on the major light purple lineage in Figure 3 (named #1 in Figure 3a,b). This lineage included a set of 21 haplotypes widely distributed (Figure 5a,b,c) in Transbaikalia - Kamchatka - Bering Sea (*h17*, *h18*, *h21*, *h32*, *h33*), Quebec - Taimyr - Chucotka - Fennoscandia (*h1*, *h2*, *h13,* *h14*, *h15*, *h16*), and in Lake Tinnsjøen (*h3*-*h13*). Based on these results, it seems plausible to evaluate that the ancestors of this major lineage colonized a large geographic area throughout the Holarctic, where some ancestral individuals also colonized Lake Tinnsjøen. Here, the founders of Lake Tinnsjøen could potentially have carried the *h1* haplotype (clade I), subsequently giving rise to clade II (*h5*, *h7*, *h8*, *h9*, *h11*, *h12*) and clade III (*h3*, *h4*) (Table 5, Figure 5).

In our study, considering the major lineage 1 (light purple; named #1 in in Figure 5a,b) with 21 haplotypes (*h1*-*h18*, *h21*, *h32*, *h33*), it is interesting to see what species designations are given to haplotypes as these should be the closest relatives to morphs in Lake Tinnsjøen. This lineage was distributed in Quebec - Bering Sea - Kamchatka - Chucotka - Transbaikalia - Taimyr - Fennoscandia, and Lake Tinnsjøen. The taxa described were: *S. alpinus* (*h1-h13*), *S. a. alpinus* (*h14-h15), S. alpinus* and *S. a. oquassa* (*h16), S. alpinus*, *S. boganidae* and *S. a. erythrinus* (*h17), S. malma and S. m. malma (h18), S. malma (h21),* and *S. a. erythrinus* (*h32, h 33)*. Based on Osinov et al. (2015), which used mtDNA CytB to assess phylogenetic relationship among taxa in *Salvelinus*, it seems that *S. alpinus*, *S. a. oquassa, S. a. erythrinus and S. malma* group together (MP tree), being genetically related to *S. boganidae* (evaluated from lack of bootstrap support). Thus, our results seems concordant with Osinov et al. (2015), and other studies regarding a genetic relationship between *S. malma* and *S. alpinus* (Horreo, 2017; Lecaudey et al., 2018; Shedko et al., 2013; Yamamoto et al., 2014), comprising taxonomic members in lineage 1 (light purple; named #1 in in Figure 5a,b).

In general, there were few similar CytB sequences in GenBank, and specifically in regard to the Fennoscandian area (as revealed in Figure 5). However, we contrast our findings with the large scale phylogeographical study by Brunner et al. (2001) who targeted Holarctic *Salvelinus* spp. using the mtDNA control region. They found five major phylogeographic lineages, named; Atlantic, Acadia, Siberia, Bering, and Arctic groups in the *Salvelinus sp.* complex. Our phylogeographic data has much less geographical coverage than Brunner et al. (2001), however, with some putative similarities. As such, our lineage 2 (yellow; named #2 in in Figure 5) may potentially fit with their Bering lineage, our lineage 5 (green; named #5 in in Figure 5) may fit with their Arctic lineage, and our lineage 1 (light purple; named #1 in in Figure 5) may fit with their Atlantic lineage. However, our lineage 1 seems to extend further north and east than the Atlantic lineage revealed by Brunner et al. (2001). A study by Gordeeva et al. (2018) studying Arctic charr (mtDNA control region) in the European part of Russia and Siberia revealed that the Atlantic group was distributed all the way to Taimyr (see also Radchenko, 2004). Alekseyev et al. (2009) found no strong support for a separation of Atlantic and Siberian haplotypes into two distinctive groups when analyzing Arctic charr in Siberia using the mtDNA control region. Thus, these studies seem to support our inference of a widespread Atlantic mtDNA lineage (Figure 5). However, we seem to lack the Siberian, the Acadian, and the Svet lineage as being revealed by Brunner et al. (2001). The discrepancy between our results and Brunner et al. (2001) may be due to lower geographical coverage, use of CytB as a much less powerful marker for divergence than the control region, and also a different set of individuals that may, or may not, belong to different *Salvelinus* taxa proposed.

REFERENCES

Alekseyev, S. S., Bajno, R., Gordeeva, N. V., Reist, J. D., Power, M., Kirillov, A. F., Samusenok, V. P., & Matveev, A. N. (2009). Phylogeography and sympatric differentiation of the Arctic charr *Salvelinus alpinus* (L.) complex in Siberia as revealed by mtDNA sequence analysis. Journal of Fish Biology, 75, 368-392. doi.org/10.1111/j.1095-8649.2009.02331.x.

Brunner, P. C., Douglas, M. R., Osinov, A., Wilson, C. C., & Bernatchez, L. (2001).

Holarctic phylogeography of arctic charr (*Salvelinus alpinus* L.) inferred from mitochondrial DNA sequences. Evolution, 55, 573-586. doi.org/10.1111/j.0014-3820.2001.tb00790.x

Consuegra, S., Elgan, J., Verspoor, E., & Garcia de Leaniz, C. (2018). Patterns of

natural selection acting on the mitochondrial genome of a locally adapted fish species. *Genetics Selection Evolution*, 47, 58. doi.org/10.1186/s12711-015-0138-0

Gordeeva, N. V., Alekseyev, S. S., Kirillov, A. F., Vokin, A. I., & Samusenok, I. V. (2018). Distribution, composition and relationships of phylogenetic groups of Arctic charr *Salvelinus alpinus* (L.) (Salmoniformes, Salmonidae) in the European part of Russia and in Siberia as revealed by the analysis of nucleotide sequences of mitochondrial DNA. *Journal of Ichyology*, 58, 808-818.

Horreo, J. L. (2017). Revisiting the mitogenomic phylogeny of Salmoninae: new insigths thanks to recent sequencing advances. *PeerJ*, Sep18:5:e3828. doi 10.7717/peerj.3828

Lecaudey, L. A., Schliewen, U. K., Osinov, A. G., Taylor, E. B., Bernatchez. L., & Weiss, S. J. (2018). Inferring phylogenetic structure, hybridization and divergence times within Salmoninae (Teleostei: Salmonidae) using RAD-sequencing. *Molecular Phylogenetics and Evolution*, 124, 82-99. doi.org/10.1016/j.ympev.2018.02.022

Osinov, A. G., Senchukova, A. L., Mugue, N. S., Pavlov, S. D., & Chereshnev, I. A. (2015). Speciation and genetic divergence of three species of charr from ancient Lake El´gygytgyn (Chukotka) and their phylogenetic relationships with other representatives of the genus *Salvelinus*. Biological Journal of the Linnean Society, 116, 63-85. doi.org/10.1111/bij.12559

Radchenko, O. A. (2004). Variability of nucleotide sequences of mitochondrial DNA Cytochrome b gene in chars of the genus *Salvelinus*. Russian Journal of Genetics, 40, 244-254. doi.org/10.1023/B:RUGE.0000021623.47492.1c

Shedko, S. V., Miroschnichenko, I. L., & Nemkova, G. A. (2013). Phylogeny of salmonids (Salmoniformes: Salmonidae) and its molecular dating: analysis of mtDNA data. *Russ Journal of Genetics*, 49, 623-637. doi.org/10.1134/S1022795413060112

Yamamoto, S., Maekawa, K., Morita, K., Crane, P. A., & Oleinik, A. G. (2014). Phylogeography of the salmonid fish, Dolly Varden *Salvelinus malma*: multiple glacial refugia in the north pacific rim. Zoological Science, 31, 660-670. doi: 10.2108/zs130266.
